# Development of vanadium-based polyanion positive electrode active materials for high-voltage sodium-based batteries

**DOI:** 10.1038/s41467-022-31768-5

**Published:** 2022-07-14

**Authors:** Semyon D. Shraer, Nikita D. Luchinin, Ivan A. Trussov, Dmitry A. Aksyonov, Anatoly V. Morozov, Sergey V. Ryazantsev, Anna R. Iarchuk, Polina A. Morozova, Victoria A. Nikitina, Keith J. Stevenson, Evgeny V. Antipov, Artem M. Abakumov, Stanislav S. Fedotov

**Affiliations:** 1 Skoltech Center for Energy Science and Technology, Skolkovo Institute of Science and Technology, 121205 Moscow, Russian Federation; 2 Department of Chemistry, Lomonosov Moscow State University, 119991 Moscow, Russian Federation

**Keywords:** Materials science, Energy storage, Batteries, Solid-state chemistry

## Abstract

Polyanion compounds offer a playground for designing prospective electrode active materials for sodium-ion storage due to their structural diversity and chemical variety. Here, by combining a NaVPO_4_F composition and KTiOPO_4_-type framework via a low-temperature (e.g., 190 °C) ion-exchange synthesis approach, we develop a high-capacity and high-voltage positive electrode active material. When tested in a coin cell configuration in combination with a Na metal negative electrode and a NaPF_6_-based non-aqueous electrolyte solution, this cathode active material enables a discharge capacity of 136 mAh g^−1^ at 14.3 mA g^−1^ with an average cell discharge voltage of about 4.0 V. Furthermore, a specific discharge capacity of 123 mAh g^−1^ at 5.7 A g^−1^ is also reported for the same cell configuration. Through ex situ and *operando* structural characterizations, we also demonstrate that the reversible Na-ion storage at the positive electrode occurs mostly via a solid-solution de/insertion mechanism.

## Abstract (web summary)

The development of high-capacity and high-voltage electrode materials can boost the performance of sodium-based batteries. Here, the authors report the synthesis of a polyanion positive electrode active material that enables high-capacity and high-voltage sodium battery performance.

## Introduction

In 1991, lithium-ion batteries (LIBs) have historically graced the electronic industry setting off a new paradigm for developers, designers, and manufacturers of portable devices. Thirty years later, the impact of the LIB technology was eventually hallmarked with a long-awaited Nobel prize^1^, though its overall contribution to our everyday life will hardly be reasonably estimated over many years to come^2,3^. Nowadays LIBs fiercely rushed into larger-scale sectors of electromotive vehicles and stationary storage systems sparking provocative and rigorous debates over the abundance of Li resources to meet the demand of emerging applications. Indeed, lithium is a scattered metal, with key mines being few and geographically isolated such that the attention of the scientific community was brought to the next alkali metal, sodium, which is hugely available with plentiful and evenly distributed resources worldwide^4–8^.

Technology-wise, the transfer to sodium-ion batteries (SIBs) does not seem capital-intensive from a first glance, because those were supposed to preserve the conventional metal-ion battery architecture^8–10^. Similar to the Li-counterparts, prospective candidates for the positive electrode (cathode) materials are Na-based transition metal oxides^11,12^ with layered structures, and polyanion-based materials maintaining rigid polyhedral frameworks that ensure higher safety and better cyclability. Among the latter, a particular courtesy was paid to vanadium-based phosphates. The NASICON family with Na_3_V_2_(PO_4_)_3_ as the most prominent representative was thoroughly examined as high-power medium-voltage cathode materials (3.4 V vs. Na^+^/Na). Afterward, Na_3_V_2_(PO_4_)O_2-x_F_1+x_ fluoride phosphates adopting the α-Na_3_Al_2_(PO_4_)_2_F_3_^13,14^ structure were proposed as high-energy materials operating at a nearly 4 V average potential. Numerous studies on both classes of materials announced delivering specific capacities close to the theoretical values with a steady performance at high charge/discharge rates with additional encouragement coming from the possibility to extract the third Na equivalent per formula unit possibly giving a further boost to the specific energy^15–19^. However, a lot of efforts were spent to deinsert all Na equivalents from Na_3_V_2_(PO_4_)_3_ and related materials, but no tangible results were achieved^15,19–21^. Contrarily, in case of Na_3_V_2_(PO_4_)O_2-x_F_1+x_ the reversible extraction of third Na can seemingly be realized by expanding the working potentials window to 1.0–4.8 V vs. Na^+^/Na at the cost of structural and cycling stability^18^. Therefore, the third sodium atom mostly serves as “dead weight” in these materials essentially diminishing their specific capacity. From this point of view, a more attractive formula would be NaVPO_4_F with one Na equivalent per one *d*-metal cation resulting in a theoretical specific capacity of 143 mAh g^−1^ being more than 10% higher than that for Na_3_V_2_(PO_4_)O_2-x_F_1+x_.

Although two polymorphs with the tetragonal (S.G. *I*4/*mmm*) and monoclinic (S.G. *C*2/*c*) structures were reported for the “NaVPO_4_F” compounds^22–24^, a closer look at their X-ray diffraction patterns and electrochemical data discloses their actual belonging to either Na_3_Al_2_(PO_4_)_2_F_3_^25–27^ or Na_3_V_2_(PO_4_)_3_^28–30^ (NASICON) structural type. It was shown in detail that a direct solid-state synthesis of NaVPO_4_F from a 1:1 mixture of VPO_4_ and NaF results in either Na_3_V_2_(PO_4_)_2_F_3_ or Na_3_V_2_(PO_4_)_3_ due to the pyrohydrolysis with the HF formation and/or VF_3_ volatilization at elevated temperatures^31,32^. However, the truly single-phase monoclinic NaVPO_4_F with the tavorite-type structure (further referred to as t-NaVPO_4_F) can be prepared via a hydrothermal route^33^. In contrast to its Li-containing counterpart t-LiVPO_4_F typically showing good electrochemical performance^34^, t-NaVPO_4_F revealed poor electrochemical activity attributed to high-energy barriers for Na^+^ diffusion in the tavorite-type framework^33,35^. A similar picture was observed for the sulfate-based analog, t-NaFeSO_4_F^36^, thus questioning the applicability of the tavorite structural type for designing cathode materials for SIBs.

Recently, a family of materials with the AMPO_4_X (A=(Li,K), K; M=V, Ti; X=O, F) general formula and a KTiOPO_4_-type (KTP) structure was discovered, showing much promise as high-power and high-voltage electrode materials for not only LIBs but also for potassium-ion batteries (PIBs)^37–43^. Here comes a reasonable question of whether the NaVPO_4_X stoichiometry can be stabilized within this KTP-type framework. In this paper, we propose a simple, efficient, and scalable synthesis approach for stabilizing NaVPO_4_F in the KTP structural type and demonstrate its practical application as a positive electrode active material for Na-ion storage. The developed cathode material is tested in a coin-cell configuration using Na metal or pre-sodiated hard carbon-based negative electrodes and a NaPF_6_-based non-aqueous electrolyte solution. In both cases, the positive electrode enables the delivery of high specific discharge capacities at various specific currents with relatively high average cell discharge voltage. Finally, via ex situ and *operando* structural characterizations, we demonstrate that the reversible Na-in storage at the positive electrode operates mostly via a solid-solution de/insertion mechanism.

## Results and discussion

**Fig. 1:**
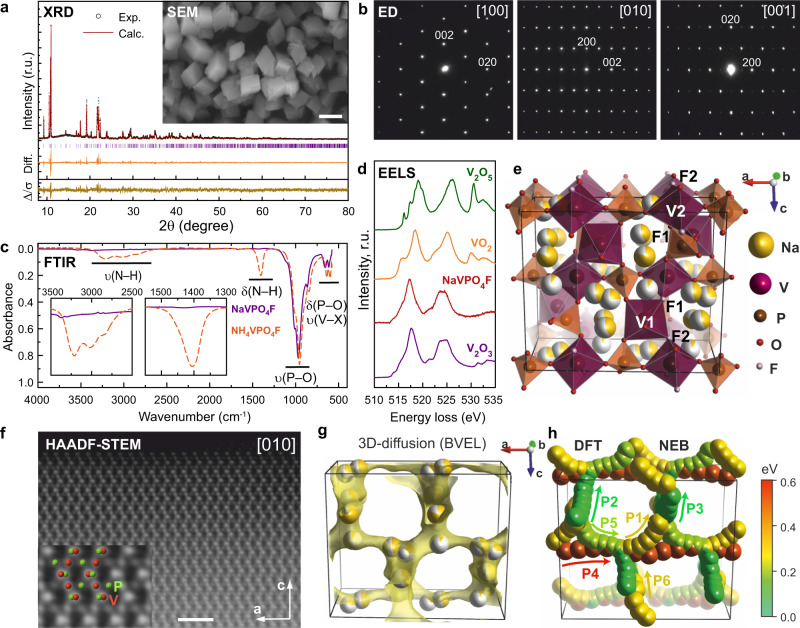
Crystal structure, composition, and Na^+^ migration pathways for NaVPO_4_F. **a** Experimental, calculated, difference and Δ/σ plots for Rietveld refinement of the NaVPO_4_F structure from SXRD data (*λ* = 1.03257 Å). Inset: SEM image of NaVPO_4_F (1 μm scale bar). **b** ED patterns of NaVPO_4_F. **c** FTIR spectra of NH_4_VPO_4_F and NaVPO_4_F in the 4000–575 cm^−1^ range. **d** EELS spectra of NaVPO_4_F and reference vanadium oxides at the V-L_2,3_ edge. **e** Ball-polyhedral representation of the NaVPO_4_F crystal structure. **f** [010] HAADF-STEM image of NaVPO_4_F (the crystal structure is overlaid), (2 nm scale bar). **g** The isosurface constructed at E_act_ of 1 eV demonstrating the 3D character of the migration pathways available for Na^+^ ions by BVEL. **h** Illustration of the considered Na–Na migration paths by DFT-NEB and their energetics in the fully inserted phase.

A pristine NaVPO_4_F fluoride phosphate has been obtained in a single step from a hydrothermally prepared NH_4_VPO_4_F precursor^44^ (Supplementary Fig. 1) by reacting with a Na glutamate excess at low temperature (190 °C, see “Methods”). The as-synthesized samples of NaVPO_4_F were identified as single-phase without impurities. The synchrotron X-ray powder diffraction (SXRD) pattern of NaVPO_4_F was fully indexed using an orthorhombic unit cell with *a* = 12.7547(4) Å, *b* = 6.2716(2) Å, *c* = 10.6400(3) Å, and V = 851.12(5) Å^3^ (Fig. 1a). The *Pna*2_1_ space group was confirmed by electron diffraction (ED) (Fig. 1b) showing 0*kl*: *k* + *l* = 2n, *h*0*l*: *h* = 2n reflection conditions. According to scanning electron microscopy (SEM), the NaVPO_4_F powder consists of well-shaped blocky rhombohedral particles of 0.6–0.8 μm in the longest dimension (Fig. 1a, inset and Supplementary Fig. 2).

The chemical composition of NaVPO_4_F was confirmed by a set of spectroscopic techniques. The Fourier-transformed infrared (FTIR) spectra of NH_4_VPO_4_F and NaVPO_4_F are compared in Fig. 1c. Importantly, NaVPO_4_F reveals no sign of stretching or bending modes intrinsic in NH_4_^+^ at 1515–1305 cm^−1^ and at 3370–2680 cm^−1^ compared to NH_4_VPO_4_F revealing intense NH_4_^+^ signals in these ranges. Moreover, NaVPO_4_F demonstrates an absence of crystal and surface H_2_O or structural OH^−^ groups, potentially replacing fluorine, since no characteristic bands in the 4000–3000 cm^−1^ and 1600–1400 cm^−1^ regions are detected. The 1200–875 cm^−1^ spectral region contains absorption bands corresponding to the stretching vibrations of the PO_4_ groups, whereas the overlapping bands located at 800–600 cm^−1^ correspond to the X–V–X (X=O, F) stretching vibrations and O–P–O bending vibrations. The Na:V:P ratio was determined by the energy-dispersive X-ray analysis in a transmission electron microscope (TEM-EDX) to be 1.01(5):0.98(6):1.01(4) which is in agreement with the ICP-AES data of 0.97(1):1.00(1):0.99(1) being close to 1:1:1 and corroborating well with the ascribed chemical formula (Supplementary Fig. 3). The TEM-EDX analysis also clearly confirms the presence of fluorine in NaVPO_4_F. The determined F content by potentiometric titration using a fluoride-ion-selective electrode corresponds to 0.9 ± 0.1 which well agrees with the formula. The 3+ oxidation state for vanadium is validated by electron energy-loss spectroscopy (EELS) (Fig. 1d) being also in line with the electron paramagnetic resonance (EPR) measurements (Supplementary Fig. 4).

Crystallographic parameters, atomic positions, and selected interatomic distances obtained with the Rietveld refinement from SXRD data (Fig. 1, a, *R*_*F*_ = 5.07%, *R*_*p*_ = 7.73%, *R*_*wp*_ = 9.78%, GOF = 1.45) are presented in Supplementary Tables 1, 2 and Supplementary Fig. 5. NaVPO_4_F adopts a KTiOPO_4_-(KTP)-type structure built upon helical chains of corner-shared VO_4_F_2_ octahedra linked by PO_4_ tetrahedra through vertexes to form a robust framework (Fig. 1e) that encloses a 3D system of intersecting cavities accommodating Na^+^ ions. The unit cell volume is ~2.2% and ~4.4% smaller than that of the potassium-containing KVPO_4_F analog and NH_4_VPO_4_F precursor, respectively, due to a smaller ionic radius of Na^+^ compared to those of K^+^ and NH_4_^+^. As in all KTP-type fluoride phosphates, the fluorine atoms occupy two *cis*- and *trans*-positions in the vicinity of the V1 and V2 atoms, respectively (Fig. 1e). Average V–X (X = O, F) bond lengths are 2.00(1) Å for the V1 site and 1.97(1) Å for the V2 site being both characteristic of the V^3+^ coordination environment^45^ (calculated bond valence sums are 2.93(6) and 3.15(6) for the V1 and V2 sites, respectively).

NaVPO_4_F demonstrates a significant disorder of Na ions with the smallest distance of around 1 Å between the split sites. The Na ions are distributed over a series of partially occupied positions forming a 3D network in the tunnels of the “VPO_4_F” framework (Fig. 1e). Such distribution might indicate high mobility of Na^+^ ions via a simple hopping mechanism. The [010] high-angle annular dark-field scanning transmission electron microscopy (HAADF-STEM) image (Fig. 1f) and [001] and [110] HRTEM images (Supplementary Fig. 6) also validate the refined structure. In the HAADF-STEM image, the bright dots correspond to the projections of V, P, and mixed V–P columns. The Na columns are virtually invisible due to the above-mentioned strong positional disorder violating the electron channeling. The specific lattice fringes on the HRTEM images are indicated.

The NaVPO_4_F framework encloses three types of channels, which might be suitable for Na^+^ migration. The topology and dimensionality of the migration system and migration activation energies were estimated using the Bond valence energy landscapes (BVEL) approach. It demonstrates the presence of continuous pathways penetrating through the entire structure in all three dimensions (Fig. 1g). This 3D Na^+^ migration pattern is different from that of the K^+^ ions in the potassium-based KTP-type KMPO_4_F (M = Ti, V) materials where helical 1D channels weaves around the crystallographic *c* direction^46–48^. To study the diffusion channels in more detail we calculated migration barriers with the nudged elastic band method within the density functional theory framework (DFT-NEB) for six independent paths (P1–P6, Fig. 1h). The energy profiles for migration paths (Supplementary Fig. 7) demonstrate that a percolating diffusion is allowed along the a and b directions with only 0.3 and 0.1 eV migration barriers, respectively. After creating one Na^+^ vacancy per unit cell in NaVPO_4_F the energy minima are not located at or close to the initial Na crystallographic sites but become more evenly distributed along the migration paths, which agrees with the experimentally observed pronounced disorder of Na positions. The migration along the *b* axis becomes slightly hindered in course of Na extraction, while the hopping along the *a* and *c* directions gets easier. This emphasizes that NaVPO_4_F should demonstrate good ion mobility in the whole range of Na concentrations, which is confirmed further by diffusion coefficient measurements and rate capability tests. Additional information and details on diffusion barriers are provided in Supporting information (Supplementary Note 1 and Supplementary Figs. 7 and 8).

**Fig. 2:**
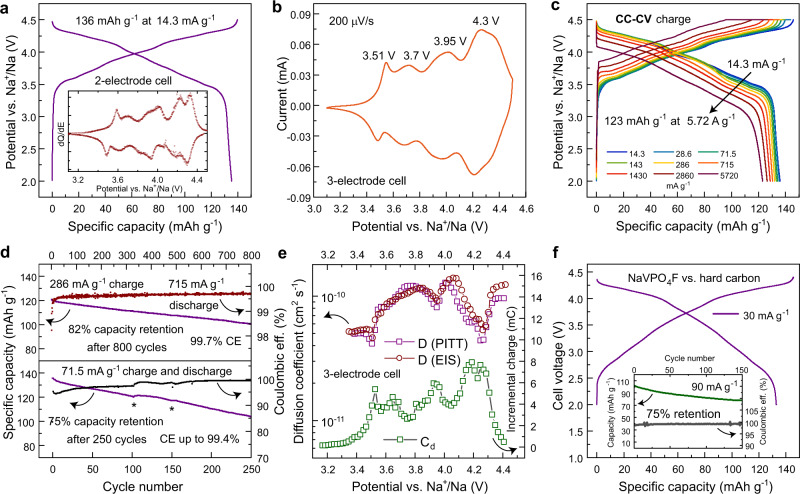
Electrochemical characterizations of the NaVPO_4_F-based electrodes in various cell configurations. **a** Galvanostatic charge/discharge curves obtained in a two-electrode Na||NaVPO_4_F cell at 14.3 mA g^−1^ rate in the 2.0–4.5 V vs. Na^+^/Na potential range. Inset: dQ/dE plot of the 2nd galvanostatic cycle at 14.3 mA g^−1^. **b** CV of NaVPO_4_F in a three-electrode Na||NaVPO_4_F cell in the 3.1–4.5 V vs. Na^+^/Na potential range at 0.2 mV s^−1^. **c** Galvanostatic rate capability measurements in the 2.0–4.5 V potential range in a constant-current + constant-voltage (CC+CV) mode, 1 C is equal to 143 mA g^−1^ (constant voltage was applied until the current reaches 14.3 mA g^−1^), two-electrode cell Na||NaVPO_4_F. **d** Top: discharge capacities during extended cycling at 715 mA g^−1^ discharge rate (286 mA g^−1^ charge rate) for 800 cycles and corresponding coulombic efficiency. Bottom: discharge capacities during extended cycling at 71.5 mA g^−1^ charge and discharge rate for 250 cycles and corresponding coulombic efficiency. The star sign marks electrolyte refreshments (see “Electrochemical measurements” section for details). **e** Diffusion coefficients as determined from PITT and EIS measurements and incremental charges of NaVPO_4_F electrode in the 3.4–4.4 V range. Measurements are done in three-electrode Na||NaVPO_4_F cells. **f** Galvanostatic charge/discharge curves for a HC||NaVPO_4_F cell consisting of a NaVPO_4_F cathode and a pre-sodiated HC anode, at 90 mA g^−1^ in the 2.0–4.4 V potential range. The specific capacity is normalized to the NaVPO_4_F electrode mass. Inset: extended cycling of the HC||NaVPO_4_F cell at 30 mA g^−1^ rate for 150 cycles. In all cases, the electrolyte used is (1 M NaPF_6_ in EC:PC:FEC (47.5:47.5:5 vol.). All measurements were conducted at 22 ± 1 °C.

The electrochemical energy storage performance of the carbon-coated NaVPO_4_F was evaluated in three- and two-electrode coin-type cells with metallic Na as a counter electrode. In both cases, it shows sloped galvanostatic curves with an average electrode potential around 4.0 V at 14.3 mA g^−1^ (1C is equal to 143 mA g^−1^ for the active material) that implies a solid-solution de/insertion mechanism preferable for the high-rate electrode materials, as it excludes the kinetically hindered nucleation of a new phase inside the pristine particles^49^ and slow phase boundary propagation due to buildup of stress and deformations^50,51^ (Fig. 2a). However, a short plateau-like region near 3.5 V with a ~30 mV hysteresis indicates a possible phase transition at the very beginning of charge involving deinsertion of ~0.15 Na^+^ per formula unit. The cyclic voltammogram (CV) (Fig. 2b) and differential capacity (dQ/dE) (Fig. 2a, inset) curves for the NaVPO_4_F electrodes also witness this reversible process at 3.51 V as characterized by a pair of sharp peaks with a peak-to-peak separation of 65 mV, followed by three pairs of broad symmetric peaks at ~3.7, 3.95, and 4.3 V with very low peak-to-peak separations (Fig. 2b).

The discharge capacity at 14.3 mA g^−1^ (C/10) in the 2.0–4.5 V potential range amounts to 136 mAh g^−1^ giving 95% of the theoretical specific capacity (143 mAh g^−1^). In this potential range NaVPO_4_F exhibits attractive capacity retention at increasing specific currents from 14.3 mA g^−1^ to 5.7 A g^−1^ (40 C) in the constant-current – constant-voltage (CC-CV) mode delivering around 130 mAh g^−1^ at 0.7 A g^−1^ (Fig. 2c) and more than 120 mAh g^−1^ at 5.7 A g^−1^ rate at the active material loading of ~2 mg cm^−2^. Noteworthy, increasing the active material loading to 8 mg cm^−2^ does not result in a significant deterioration of the rate performance up to 1.43 A g^−1^ (10 C), though at the 2.86 A g^−1^ (20 C) and 5.7 A g^−1^ (40 C) specific currents, the specific capacities drop-down at higher loadings (Supplementary Fig. 9). These tests anticipate that the loading can be further enlarged without sacrificing the attainable capacities. The extended cycling at a 5 C discharge rate for over 800 cycles reveals only 18% capacity fading (Fig. 2d, top). At a lower specific current of 71.5 mA g^−1^ (C/2) the capacity fading is expectedly more noticeable reaching 25% within 250 cycles (Fig. 2d, bottom). The faster capacity decay at the lower specific current of 71.5 mA g^−1^ might be explained by a consequently longer exposure of the Na||NaVPO_4_F cell to high potentials where the electrolyte instability is generally more pronounced leading to a polarization buildup and consequent performance degradation^52^.

Diffusion coefficients of Na^+^ in NaVPO_4_F (as determined from electrochemical impedance spectroscopy (EIS) and potentiostatic intermittent titration technique (PITT)) exhibit values in the range of 10^–11^−10^–10^ cm^2^ s^−1^ (Fig. 2e) exceeding those in structurally related α-VPO_4_^53^ and AVPO_4_F^38^. Notably, a pronounced minimum in the diffusion coefficient appears at around 3.5 V with a sharp rise in the differential capacitance C_d_ implying a possible two-phase transition and associated phase separation. In addition, the diffusion coefficients at the initial extraction of *~*0.15 Na^+^ are lower than the values after the anticipated phase transformation. The EIS spectra of the NaVPO_4_F electrodes show a high-frequency semicircle with a diameter of ~30 Ω mg (per mass of active material in a composite electrode) (Supplementary Fig. 10a), indicating the resistance of surface layers^54,55^. The low resistance of the surface layers to Na^+^ transport implies low reactivity of the material surface with the electrolyte. The medium frequency semicircle exhibits a pronounced variation with the potential suggesting it to be the charge transfer resistance^54,56^. The latter converges to a value of *~*20 Ω·mg active material at the potential 3.6 V (Supplementary Fig. 10b) indicating fast ion transfer kinetics and ensuring low kinetic polarization during the cathode operation.

Further, the HC||NaVPO_4_F cells with the NaVPO_4_F cathode and pre-sodiated hard carbon (HC) anode were assembled to demonstrate the electrochemical performance of the 4 V Na-based electrochemical system. The electrochemical performance of HC in combination with a Na metal electrode is presented in Supplementary Fig. 11. Figure 2f illustrates the galvanostatic charge/discharge curves of the HC||NaVPO_4_F cell having a capacity ratio of positive to negative electrodes of 1:1 at 60 mA g^−1^ and 30 mA g^−1^ charge/discharge rates. The cell operates at the average voltage of 3.7 V delivering more than 130 mAh g^−1^ specific capacity (recalculated to the active mass of NaVPO_4_F) at 30 mA g^−1^ (at 23 °C). Noteworthy, the cell performance is dependent also on the performance of the corresponding anode material that mainly contributes to capacity fading at extended cycling. At 90 mA g^−1^rate, the HC||NaVPO_4_F retains 75% of the initial capacity after 150 cycles (Fig. 2f, inset), while the Na||NaVPO_4_F cell is capable of sustaining hundreds of cycles preserving more than 80% of the specific capacity (Fig. 2d, inset). Unlocking the full potential of the NaVPO_4_F cathode material requires the development of stable high-power anode material.

**Fig. 3:**
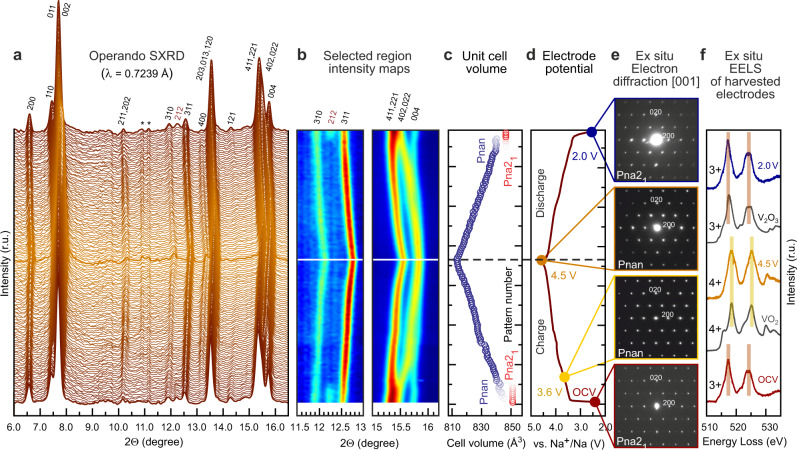
Structural evolution and charge compensation mechanism of NaVPO_4_F during cycling. **a** *Operando* SXRD diffraction patterns in the 6.0–16.5° 2Θ range (*λ* = 0.7239 Å). The SXPD pattern highlighted in bold shows the charged phase. The asterisk sign (*) designates the reflections belonging to coin-cell components. **b** Magnified regions of the *operando* SXRD intensity map. Horizontal full white and dashed black lines show the charged phase. **c** Cell volume evolution on charge/discharge as refined from the operando SXRD data. Error bars are within the circles. Major ticks mark every 20th pattern. **d** Corresponding charge-discharge profile in the Na||NaVPO_4_F cell, 1 M NaPF_6_ in EC:PC:FEC (47.5:47.5:5 by vol.) electrolyte, the second cycle, measurements were conducted at 22 ± 2 °C. **e** [001] ED patterns for the pristine material and recovered electrodes at 3.6, 4.5, and 2.0 V showing (dis)appearance of *hk*0: *h* + *k* = 2n + 1 reflections corresponding to the *Pna*2_1_↔*Pnan* phase transition. **f** Ex situ EELS spectra for the harvested electrodes at OCV, charged to 4.5 V, and discharged to 2.0 V manifesting the reversible change of vanadium oxidation state. The V_2_O_3_ and VO_2_ spectra are given for reference.

The phase transformations and related changes in the unit cell parameters in NaVPO_4_F during Na de/insertion were examined with *operando* SXRD (Fig. 3a–d).

The SXRD patterns reveal that Na^+^ deinsertion from NaVPO_4_F is reversible with the reflections almost fully returning to their initial positions and recovering intensities. Negligibly small volume change of 0.5% between the initial and cycled phases can be attributed to an insufficient discharge depth achieved at the cut-off potential of 2.5 V vs. Na^+^/Na. The charge and discharge processes can be considered reversible since no significant difference between the patterns on charge and discharge can be observed. The corresponding reversible contraction of the cell volume on charge amounts to ~4.5%. Peculiar changes at 3.5–3.6 V corresponding to the specific feature on the CV curves and PITT data described above, hint at a two-phase transformation presumably associated with Na^+^ ion (re)ordering (Fig. 3b). A similar peculiarity with the 212 reflection disappearing was observed for KVPO_4_X (X=O, F)^39^, and in KTiPO_4_F (related to a monoclinic distortion of *Pna*2_1_ to *Cm*)^47^. In case of NaVPO_4_F, at these potentials with extraction of ~0.15 Na^+^ the orthorhombic symmetry preserves and a reversible *Pna*2_1_-to-*Pnan* transition occurs as deduced from the analysis of the ED patterns for the recovered electrodes (Fig. 3e and Supplementary Fig. 12). However, from the structural viewpoint, this transformation is supposed to be minor since the polyhedral framework does not undergo pronounced distortions and the difference in the unit cell volumes between the two phases is less than 0.8% (Fig. 3d). For x > 0.15 the *Pnan* Na_1-x_VPO_4_F operates via a single solid-solution regime, as also confirmed with a convex hull calculation using DFT+U and cluster expansion methods (Supplementary Fig. 13).

To investigate the charge compensation mechanism in NaVPO_4_F the vanadium oxidation state evolution during the extraction/insertion of Na^+^ was studied via ex situ EELS measurements carried out on harvested electrodes charged to 4.5 V and discharged to 2.0 V after full charge to 4.5 V vs. Na^+^/Na (Fig. 3f). A pronounced shift towards higher energy loss is observed in the V-L_2,3_ edge for the electrode charged to 4.5 V vs. Na^+^/Na, which is related to the increasing vanadium oxidation state from 3+ to 4+. At 2.0 V, both intensities and positions of the L_2_ and L_3_ peaks recover their initial values that are characteristic of V^3+^ thus validating a reversible V^3+^↔V^4+^ transition in NaVPO_4_F.

**Fig. 4:**
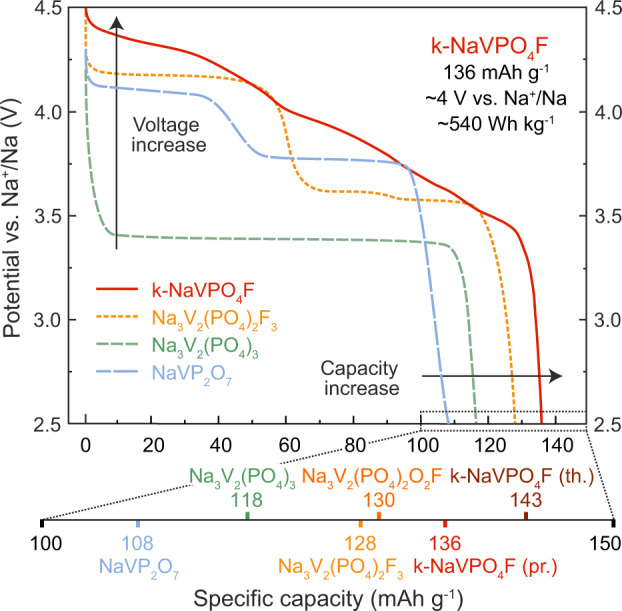
Voltage profiles for vanadium-based positive electrodes. Potential vs. specific capacity plots for Na_3_V_2_(PO_4_)_3_, Na_3_V_2_(PO_4_)_2_F_3_, β-NaVP_2_O_7_ normalized to the theoretical specific capacity per one-electron V^3+^↔V^4+^ transition from experimental data^59–61^. For k-NaVPO_4_F, the experimental discharge profile is presented for comparison. For all the materials, the theoretical specific capacity values are reported.

In conclusion, we have developed and synthesized the k-NaVPO_4_F positive electrode material for sodium-ion batteries. In contrast to previously known electrochemically inactive tavorite-type t-NaVPO_4_F^32,33,57^, k-NaVPO_4_F with the KTiOPO_4_-type structure demonstrates attractive specific capacity of 136 mA h g^−1^ at 14.3 mA g^−1^ specific current, high C-rate performance stemming from low energy barriers and high diffusion coefficients, and extended cycling stability. Worth noting that this material delivers practical specific capacity of 136 mAh g^−1^ at 14.3 mA g^−1^ that is higher than the theoretical capacity of the commercialized Na_3_V_2_(PO_4_)_2_F_3_ (128 mAh g^−1^)^18,58,59^ as well as other known vanadium-based positive electrodes for one-electron V^3+^↔V^4+^ transition (Fig. 4), such as NASICON-type Na_3_V_2_(PO_4_)_3_^15,20,21^, Na_3_V_2_(PO_4_)_2_F_3_ (or its closest relative, Na_3_V_2_(PO_4_)_2_O_2_F), β-NaVP_2_O_7_^60^, etc. The V^4+^/V^3+^ redox potential in this material achieves 4.3 V vs. Na^+^/Na which is among the highest values for vanadium-based positive electrodes. This increase in potential in comparison to other closely related phosphates and fluoride phosphates might stem from the KTP-type structure peculiarities as also shown for Ti-containing materials adopting the same KTP structure and displaying high electrode potentials^47^. Moreover, among the vanadium-based positive electrodes with close specific capacity values, k-NaVPO_4_F is the only one that operates mostly via solid-solution Na-storage mechanism, while others experience single or multiple two-phase transformations which are also reflected by their plateau-like discharge voltage profiles (Fig. 4).

The full potential of the k-NaVPO_4_F cathode material is yet to be delivered which would further boost the achievable specific energy of next-generation sodium-ion batteries. Overall, this study validates that following the solid-state chemistry guidelines and principles when choosing a proper combination of chemical composition, crystal structure, and synthesis method, enables producing inorganic materials with appealing electrochemical properties.

## Methods

### Synthesis

NaVPO_4_F was synthesized by means of a solid-state ion-exchange approach in two steps. First, a NH_4_VPO_4_F precursor was obtained using a hydrothermal route according to the reaction (1). For this, sodium carboxymethylcellulose (0.200 g) was dissolved in distilled H_2_O (22 ml) under heating with vigorous stirring. The resulting solution was cooled down to room temperature. Then VOSO_4_ × 3H_2_O (1.0857 g, 98%, Reakhim), NH_4_H_2_PO_4_ (0.8627 g, 99% Sigma-Aldrich), (NH_4_)_2_HPO_4_ (0.9905 g, 99%, Sigma-Aldrich), N_2_H_6_SO_4_ (0.2440 g, 98%, Reakhim) and NH_4_HF_2_ (0.2850 g, 99%, Reakhim) were added to the solution one after another under constant stirring until a light-blue suspension is formed. Both NH_4_H_2_PO_4_ and (NH_4_)_2_HPO_4_ are required to maintain a neutral buffer solution with pH ~7. The resulting dark-blue suspension was transferred in a 30 ml Teflon-lined stainless-steel autoclave (Parr instruments). The autoclave was sealed and heated at 200 °C for 4 h. The precipitate was collected, washed several times with deionized water and once with acetone, then dried.

1 $$4{{{{{{\rm{VOSO}}}}}}}_{4}+\, 2({{{{{\rm{NH}}}}}}_{4})_{2}{{{{{{\rm{HPO}}}}}}}_{4}+{2{{{{{\rm{NH}}}}}}}_{4}{{{{{{{\rm{H}}}}}}}_{2} {{{{{\rm{PO}}}}}}}_{4}+{2{{{{{\rm{NH}}}}}}}_{4}{{{{{{\rm{HF}}}}}}}_{2} +{{{{{{\rm{N}}}}}}}_{2}{{{{{{\rm{H}}}}}}}_{6}{{{{{{\rm{SO}}}}}}}_{4}\\ \mathop{\to }\limits^{200^\circ {{{{{\rm{C}}}}}}}{4{{{{{\rm{NH}}}}}}}_{4}{{{{{{\rm{VPO}}}}}}}_{4}{{{{{\rm{F}}}}}}+{4{{{{{\rm{NH}}}}}}}_{4}{{{{{{\rm{HSO}}}}}}}_{4}+{{{{{{{\rm{H}}}}}}}_{2}{{{{{\rm{SO}}}}}}}_{4}+{{{{{{\rm{N}}}}}}}_{2}\uparrow +{4{{{{{\rm{H}}}}}}}_{2}{{{{{\rm{O}}}}}}$$

The product was mixed with sodium glutamate (99%, Prime Chemicals Group) in a molar ratio of 1:5 and annealed at 190 °C for 10 h. The principal reaction follows:

2 $${{{{{{\rm{NH}}}}}}}_{4}{{{{{{\rm{VPO}}}}}}}_{4}{{{{{\rm{F}}}}}}+{{{{{\rm{GluNa}}}}}}\times {{{{{{\rm{H}}}}}}}_{2}{{{{{\rm{O}}}}}}\mathop{\to }\limits^{190^\circ {{{{{\rm{C}}}}}}}{{{{{{\rm{NaVPO}}}}}}}_{4}{{{{{\rm{F}}}}}}+{{{{{{\rm{H}}}}}}}_{2}{{{{{\rm{O}}}}}}\uparrow +{{{{{{\rm{NH}}}}}}}_{3}\uparrow +^{\prime\prime} {{{{{\rm{GluH}}}}}}^{\prime\prime}$$

The “GluH” here designates the remaining partly or fully decomposed organic products and other carbon residues. Importantly, the described procedure enables obtaining pristine Na-based compounds with no residual NH_4_^+^ ions. The final product was washed with hot deionized water several times. For carbon coating, NaVPO_4_F was mixed with polyacrylonitrile, PAN (M_w_ 150,000, Sigma-Aldrich) in a weight ratio of 93:7. Then dimethylformamide (DMF) was added dropwise to dissolve PAN. The resulting viscous mixture was poured into ethanol to coagulate. Then the formed sticky slurry was dried at 60 °C under vacuum and further calcined at 525 °C for 2 h under argon flow purified from traces of oxygen using metallic iron powder.

### Material characterization

X-ray powder diffraction (XRD) patterns were collected with a Guinier camera Huber G670 powder diffractometer (Co-K_α1_ radiation, 4–100° 2*θ* range). The synchrotron XRD experiment for NaVPO_4_F was performed at the MCX beamline (Elettra Sincrotrone Trieste) at a wavelength of *λ* = 1.03257 Å in a transmission geometry using a point fast scintillator detector^62^.

The Na:V:P elemental ratio was determined by inductively coupled plasma atomic emission spectroscopy (ICP-AES) using a Perkin Elmer Optima 5300 apparatus. The F content in NaVPO_4_F was determined via direct potentiometry using an ELIS-131F fluoride-ion selective electrode. Total Ionic Strength Adjustment Buffer (TISAB) was prepared by mixing 500 mL H_2_O, 57 mL glacial acetic acid (Merck), 58 g of NaCl (Sigma-Aldrich, puriss., p.a.) and 0.3 g of sodium citrate (Sigma-Aldrich, >99%). The solution volume was adjusted to 1 L. The solution was titrated to pH 5 with 5 M NaOH (Sigma-Aldrich, 50 wt.% solution in water). Fluoride standards were prepared from high-purity NaF (99.99 Suprapur). In a typical procedure, 12 mL of TISAB were added to 12 mL of fluoride standard or test solution and the volume of the flask was adjusted to 25 mL with deionized water (MilliQ, 18 MOhm). The solution was stirred, and the potential of the ion-selective electrode was determined after five minutes. The calibration curve is shown in Supplementary Fig. 14. A weighted amount of NaVPO_4+y_F_1-x_ material was heated in 30% H_2_O_2_ with magnetic stirring until a transparent solution was formed. The resulting solution was diluted to 25 mL to reach the concentration of F^−^ 5 × 10^−4^ M.

Scanning electron microscopy (SEM) analysis was done using a Quattro S scanning electron microscopy (Thermo Fisher Scientific, LaB_6_ field emission cathode) in a secondary electron mode with an accelerating voltage of 5 kV. The energy-dispersive X-ray analysis (EDX) was performed on a scanning electron microscope (SEM) JEOL JSM-6490LV (W-cathode, operating at 30 kV) equipped with an EDX system INCA Energy+ (Oxford, Si-(Li)-detector) and on Titan Themis Z transmission electron microscope at 200 kV.

For the transmission electron microscopy (TEM) studies, ~10 mg of the sample was ground with an agate mortar under ethanol. The resulting suspension was deposited onto a carbon film supported by a copper grid. Electron diffraction (ED) patterns, high-resolution transmission electron microscopy (HRTEM) images and high-angle annular dark-field scanning transmission electron microscopy (HAADF-STEM) images were recorded with an aberration-corrected Titan Themis Z transmission electron microscope at 200 kV. Electron energy-loss spectra (EELS) were collected with a Titan Themis Z transmission electron microscope operated at 120 kV and equipped with a monochromator and Gatan Quantum ER965 spectrometer.

Thermal analysis was carried out with a TG-DSC F3 STA-449 apparatus (Netzsch) combined with a mass spectrometer QMS 403 D Aëolos (Netzsch) under Ar or dry air flow (50 ml/min). The powders were heated at 10 K/min rate in the 35–900 °C temperature range. Pristine NaVPO_4_F can be considered stable in argon up to 550 °C (Supplementary Fig. 15a). The residual carbon content in the carbon-coated NaVPO_4_F/C was estimated to be ~10 wt.% which was taken into account when calculating the specific capacities (Supplementary Fig. 15b). The material undergoes a decomposition reaction at temperatures higher than 550 °C, which is clearly shown by the appearance additional reflections and bands at XRD and FTIR (Supplementary Fig. 15c, d and Supplementary note 2).

The attenuated total reflectance Fourier transform infrared (ATR-FTIR) measurements were performed with a stand-alone FTIR microscope LUMOS (Bruker) equipped with germanium ATR crystal and liquid N_2_ cooled MCT detector. Spectra were recorded in the 4000–600 cm^−1^ range with 2 cm^−1^ resolution and averaging of 64 scans. The reproducibility was checked by probing different spots of the same powder sample. The thermal behavior of NaVPO_4_F was also assessed by the temperature-dependent FTIR coupled with XRD analysis (Supplementary Fig. 13c, d).

The electron paramagnetic resonance (EPR) measurements were performed using an X-band spectrometer with a high-frequency (100 kHz) magnetic field modulation (SPIN, Russia). Spectra were recorded with 2 mW input microwave power and modulation amplitude of 0.2 mT.

### Structure analysis

The Rietveld refinement of the NaVPO_4_F structure from synchrotron X-ray powder diffraction data was performed in GSAS-II program package^63^ with the KVPO_4_F structure (S.G. *Pna*2_1_, #33) as an initial model with potassium replaced by sodium. Total Na sites occupancies were constrained to 2 (sum of two sites per asymmetric unit). Isotropic atomic displacement parameters were fixed at 0.01 Å^2^. The crystal structures were drawn using VESTA software^64^.

### Electrochemical measurements

For electrochemical testing, the NaVPO_4_F electrodes were prepared by mixing 80 wt. % of the NaVPO_4_F/C active material, 10 wt. % acetylene black (carbon Super-C) and 10 wt. % polyvinylidene fluoride (pVdF, Sigma-Aldrich). N-methyl pyrrolidinone (NMP, Sigma-Aldrich, anhydrous, 99.5%) was added to dissolve pVdF. The resulting slurry was cast on the aluminum foil (40 μm, graphitized, Tob New Energy, China) by a Doctor Blade technique at different thicknesses (100–300 μm). The electrodes were dried under vacuum at 110 °C for 12 h to remove residual NMP and cut into disks (16 mm in diameter). The active material loading after drying varied from ~2 to ~8 mg cm^−2^, the electrode thickness 15–50 μm depending on the loading and type of the further analysis. The electrochemical properties of NaVPO_4_F were evaluated using coin-type two-electrode cells with Al and Cu (15 μm, 99.9%, Tob New Energy, China) current collectors, 1 M NaPF_6_ electrolyte solution (NaPF_6_, dried at vacuum for 24 h at 25 °C, Alfa-Aesar, 99.5%) in EC:PC:FEC (47.5:47.5:5 vol., Sigma-Aldrich, anhydrous) in the amount of 70–80 μL (water content less than 20 ppm, measured by Karl Fischer titration, Mettler-Toledo, C20), glass-fiber separators (Whatman, thickness 260 μm, 1.6 μm pore size) and Na metal (Sigma-Aldrich, chunks, roll-pressed into ~100 μm foil and cut as 16 mm disks) used as both counter and reference electrodes. The galvanostatic measurements were carried out at 22 ± 1 °C using a Biologic VMP3 potentiostat. No climatic/environmental chamber was used for cycling tests, the testing room was air-conditioned at 22 ± 1 °C. For the electrolyte refreshment, the cell was disassembled in the Ar-filled glovebox, the working electrode was washed with PC and put into a new cell with a fresh Na counter electrode and a fresh portion of the electrolyte. This procedure was done to check if the performance decay is influenced by the electrolyte decomposition or material structure degradation. The specific capacity and current were calculated based on the mass of NaVPO_4_F.

Cyclic voltammetry, potentiostatic and galvanostatic intermittent titration and impedance measurements were done in three-electrode cells to ensure precise control of the working electrode potential. Three-electrode cells were stainless-steel coin-type cells with sodium disk as a counter electrode on a Cu current collector and a NaVPO_4_F/C working electrode on an Al current collector. Reference electrodes were fabricated by charging the Na_3_V_2_(PO_4_)_3_ electrodes (active material:carbon Super-C:pVdF ratio 8:1:1 by mass) to reach the state-of-charge of 0.5 as described previously^65^ and placed on a stainless-steel ring, separated from the counter and working electrodes by PTFE stoppings and glass-fiber separators. The potential of the reference electrode was 3.370 V vs. Na^+^/Na (the potential of a two-phase transition which accompanies the V^4+^/^3+^ redoх in Na_3_V_2_(PO_4_)_3_^66^). All the reported potentials were recalculated into the Na^+^/Na scale. The average equilibrium potential of NaVPO_4_F was estimated by averaging the charge and discharge potentials at a low cycling rate of 14.3 mA g^−1^ to minimize the polarization effects. The average potential was found to be 4.0 V. The measurements were done in a coin-type three-electrode cell to exclude the changes in potential fluctuations at Na metal reference electrode provoked by surface reactions with the electrolyte.

### Diffusion coefficients measurements

Diffusion coefficients for Na^+^ ions in NaVPO_4_F lattice were estimated from PITT and EIS data following the approaches detailed elsewhere^67,68^. For the analysis of both the PITT and EIS data, the spherical diffusion geometry was adopted with the diffusion length 0.5 μm as estimated from SEM data. PITT measurements were performed in 10 mV steps with the step duration of 1.5 h in the potential range 3.4–4.4 V vs. Na^+^/Na. EIS measurements were performed in the same potential interval in the frequency range 100 kHz–10 mHz with a 5 mV amplitude; 7 points per decade were registered. Prior to the EIS measurements, the electrode was polarized potentiostatically until the current reached the background value of 0.1–0.2 µA cm^−2^. Parameters of EIS fitting are given in Supplementary Table 3. The equivalent circuit for the experimental spectra fitting comprised R_CEI_/C_CEI_ (resistance and capacitance of surface layers), R_ct_/C_dl_ (charge transfer resistance and double-layer capacitance) contours and a finite spherical Warburg element (Supplementary Fig. 10b). MEISP software was used to approximate the experimental spectra to the equivalent circuit. GITT measurements were also conducted to calculate the diffusion coefficients (Supplementary Fig. 17 and Supplementary Note 3).

### Hard carbon preparation and electrode fabrication

Hard carbon (HC) anode material was synthesized by the hydrothermal method^69^. The amorphous organic precursor was obtained from a 2 M glucose solution in distilled H_2_O by treatment in a Teflon-lined autoclave (304 stainless steel) at 180 °C for 16 h. The resulting brown powder was collected and washed three times with water, and annealed in Ar flow (100 mL min^−1^) at 1200 °C within 5 h. Electrodes were fabricated by mixing the active material, carbon black (Super-C), polyvinylidene fluoride (PVDF) in the weight ratio of 80:10:10 in N-methyl-2-pyrrolidone (NMP, Sigma-Aldrich, anhydrous). The slurry was spread onto the aluminum foil (40 μm width, graphitized, MTI) with a typical HC loading after drying being 2–3 mg cm^−2^.

The electrochemical energy storage properties of HC anodes were studied by galvanostatic charge/discharge profiling in 1 M NaPF_6_ electrolyte solution (NaPF_6_, dried at vacuum for 24 h at 25 °C, Alfa-Aesar, 99.5%) in EC:PC:FEC (47.5:47.5:5 vol., Sigma-Aldrich, anhydrous), glass-fiber separators and sodium metal (Sigma-Aldrich) negative electrode. The experiments were done at various specific currents (Supplementary Fig. 11), and the extended cycling was carried out at 90 mA g^−1^ in the 0.01–2.50 V vs. Na^+^/Na potential range at 22 ± 1 °C using a Biologic VMP3 potentiostat. The specific capacity is calculated based on the mass of active material (HC).

### Na-ion cell assembly and testing

The HC|| NaVPO_4_F coin-type (CR-2032) cells were assembled with the 1:1 cathode/anode capacity ratio with pre-cycled NaVPO_4_F/C cathode and pre-sodiated HC anode electrodes to form CEI/SEI and tested in galvanostatic regime at 30 mA g^−1^ and 90 mA g^−1^ charge/discharge currents with the 1 M NaPF_6_ (Sigma-Aldrich, 99.9%) in EC:PC:FEC (47.5:47.5:5 by vol., Sigma-Aldrich, anhydrous) electrolyte and glass-fiber separators (Whatman) in the 2.0–4.4 V potential range at 22 ± 1 °C using a Biologic VMP3 potentiostat. The specific capacity (Fig. 2f) is calculated based on the mass of the active positive electrode (NaVPO_4_F).

### Preparation and ex situ measurements of the electrode sample

NaVPO_4_F electrodes for ex situ ED and EELS measurements were prepared by galvanostatic charge/discharge in Na||NaVPO_4_F stainless-steel coin-type cells to the specific cutoff potential (Fig. 3d–f and Supplementary Fig. 12). The cells were disassembled and the active electrode materials were harvested and washed with PC to remove the electrolyte residues in an Ar-filled glovebox, then deposited in a powder form onto a carbon film supported by a copper grid and transferred to the Titan Themis Z transmission electron microscope using a special vacuum-transfer holder for further ED and EELS measurements.

### Operando X-ray diffraction

Composite NaVPO_4_F/C electrodes for the *operando* synchrotron X-ray diffraction (SXRD) analysis were prepared in accordance with the above-mentioned scheme (see electrochemical measurements section, the material loading of 2–3 mg cm^−2^). The experiments were performed at MCX beamline (Elettra Sincrotrone, Trieste)^62^ at a wavelength of *λ* = 0.7293 Å in a transmission geometry using the marCCD-SX-165 2D detector. Modified CR-2032 coin-type Na||NaVPO_4_F cells with Kapton^®^-glued windows were fabricated and assembled with the NaVPO_4_F cathode material, 1 M NaPF_6_ electrolyte solution (NaPF_6_, dried at vacuum for 24 h at 25 °C, Alfa-Aesar, 99.5%) in EC:PC:FEC (47.5:47.5:5 vol., Sigma-Aldrich, anhydrous), glass-fiber separator (Whatman) and metallic Na anode (Sigma-Aldrich, chunks, roll-pressed into ~100-μm foil and cut as 16 mm disks). Before the operando experiment, the cells ran for one charge/discharge cycle at 20 mA g^−1^. The *operando* SXRD data were collected under a galvanostatic cycling with potential limitation (GCPL) experiment at 20 mA g^−1^ charge and 10 mA g^−1^ discharge rates in the potential range of 2.5–4.6 V vs. Na^+^/Na using a Biologic SP-150 potentiostat at 22 ± 2 °C. 2D and integrated 1D powder diffraction patterns were processed with GSAS-II software^63^. The unit cell fit on operando data was done in TOPAS software^70^. The phases were fitted using a parametric approach^71^. The *Pnan* phase was allowed to vary cell parameters freely, while for the *Pna*2_1_ phase the cell parameters were fitted parametrically using constant unit cell volume for the *Pna*2_1_ phase and assuming this structure does not expand/contract during charge/discharge process. Weight fraction was varied freely.

### Bond valence energy landscape (BVEL) method

The Bond Valence Energy Landscapes (BVEL) analysis of the Na^+^ migration pathways was performed using the 3DBVSMAPPER2.0 software^72^ in the VPO_4_F framework. The details and theory behind the method as well as its applications can be found elsewhere^57,73,74^.

### DFT calculations

The DFT calculations are performed using the projected augmented plane wave method, with the Vienna ab initio simulation package (VASP)^75^ and the high-throughput python-based package SIMAN^35^. We adopt generalized gradient approximation (GGA) to exchange-correlation functional and standard PAW PBE potentials^76^ with a minimum number of valence electrons. To take into account the strongly correlated character of the d-electrons of vanadium, a Hubbard-like correction is added within the Dudarev scheme^77^ and U value of 3.1 eV, which was used in our previous studies for KVPO_4_F and RbVPO_4_F^46^. Gaussian smearing with a smearing width of 0.1 eV was used for Brillouin-zone integrations. All calculations were performed with spin polarization with the ferromagnetic ground state. The energy cutoff was fixed at 400 eV, the Gamma-centered k-point was 4 × 2 × 2 for the unit cell with 64 atoms. To eliminate Pulay errors the lattice optimization (ISIF = 4) was performed at constant volume for several contracted and expanded cells (seven points).

The migration barriers were determined using nudged elastic band (NEB) method and GGA PBE without U as implemented in VASP using the 1 × 2 × 1 supercell. The method allows finding the minimum energy path, which includes several intermediate configurations (images) between initial and final states. Five intermediate images were used.

The cluster expansion method, such as implemented in the ATAT code, was used to study phase stabilities^78^. The root of the mean square error of the energies from the resulting cluster expansion Hamiltonian with respect to DFT+U energies was estimated to be less than 0.015 eV per Na ion.

The initial crystal structure of NaVPO_4_F with the *Pna*2_1_ space group was taken from the experiment. In all cases, after Na removal, full optimization of the unit cell and atomic positions were performed. The optimization was performed using a quasi-Newton algorithm until the maximum force permitted for any vector component was less than 0.1 eV Å^−1^ for NEB calculations and 0.05 eV Å^−1^ for all other calculations. The computational setup, including errors due to the periodic boundary conditions, has been estimated to provide the precision of 0.1 eV for migration barriers.

### Reporting summary

Further information on research design is available in the Nature Research Reporting Summary linked to this article.

## Supplementary information

Supplementary information

Reporting Summary

## Data Availability

All data that support the findings of this study are presented in the manuscript and in the supplementary information file. The NaVPO_4_F structural data generated in this study (crystallography information file, cif) have been deposited to CCDC database under accession code 2176463 and can be found online at 10.25505/fiz.icsd.cc2c1sh. Raw data for transmission electron microscopy and operando synchrotron X-ray diffraction are available from the corresponding author upon reasonable request. Source data are provided with this paper.

## Source data

Source data
