# Supplementary material for: Development of vanadium-based polyanion positive electrode active materials for high-voltage sodium-based batteries
Source: Nat Commun. 2022 Jul 14;13:4097. doi: 10.1038/s41467-022-31768-5 (PMC9283384; doi:10.1038/s41467-022-31768-5)
Supplement: Supplementary file 1 — Supplementary information [file 41467_2022_31768_MOESM1_ESM.pdf]

Supplementary information

Development of vanadium-based polyanion positive electrode active materials for high-voltage sodium-based batteries

*Semyon D. Shraer[1,2], Nikita D. Luchinin[1], Ivan A. Trussov[1], Dmitry A. Aksyonov[1], Anatoly V. Morozov[1], Sergey V. Ryazantsev[1,2], Anna R. Iarchuk[1], Polina A. Morozova[1], Victoria A. Nikitina[1,2], Keith J. Stevenson[1], Evgeny V. Antipov[2,1], Artem M. Abakumov[1], Stanislav S. Fedotov[1,]**

[1] Skoltech Center for Energy Science and Technology, Skolkovo Institute of Science and Technology, 121205 Moscow, Russian Federation.

[2] Department of Chemistry, Lomonosov Moscow State University, 119991 Moscow, Russian Federation.

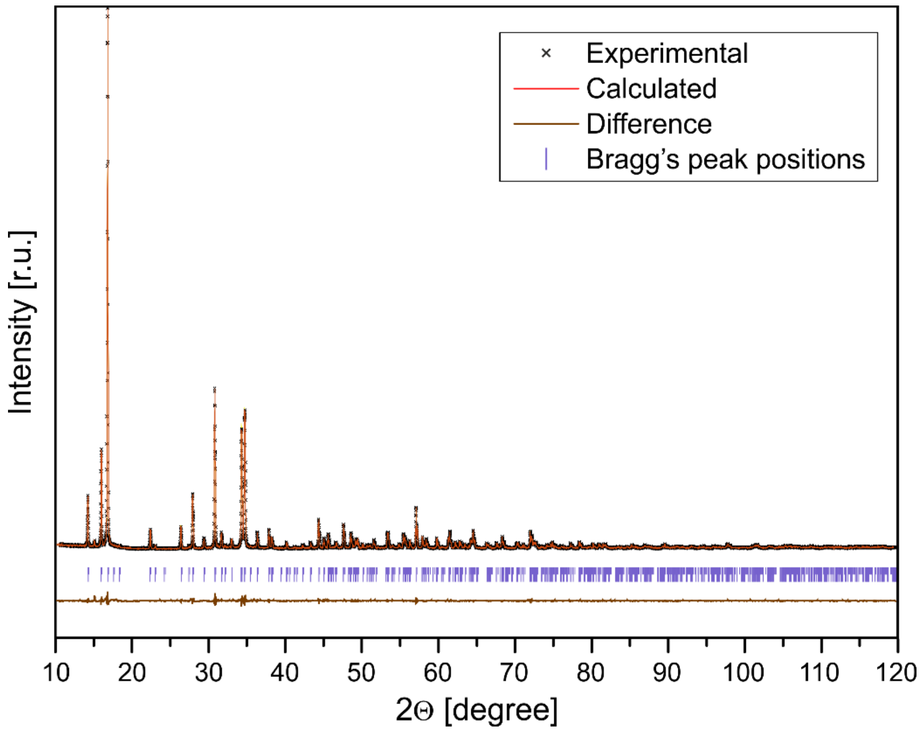

Supplementary Figure 1. Le Bail fit of X-ray powder diffraction pattern for NH_4VPO_4F with a = 12.9562(1) Å, b = 10.58622(8) Å, c = 6.49298 (4) Å (V = 890.557(8) Å^3), space group *Pnn*2, GOF = 1.18, R_p = 5.45, R_{wp} = 7.47.

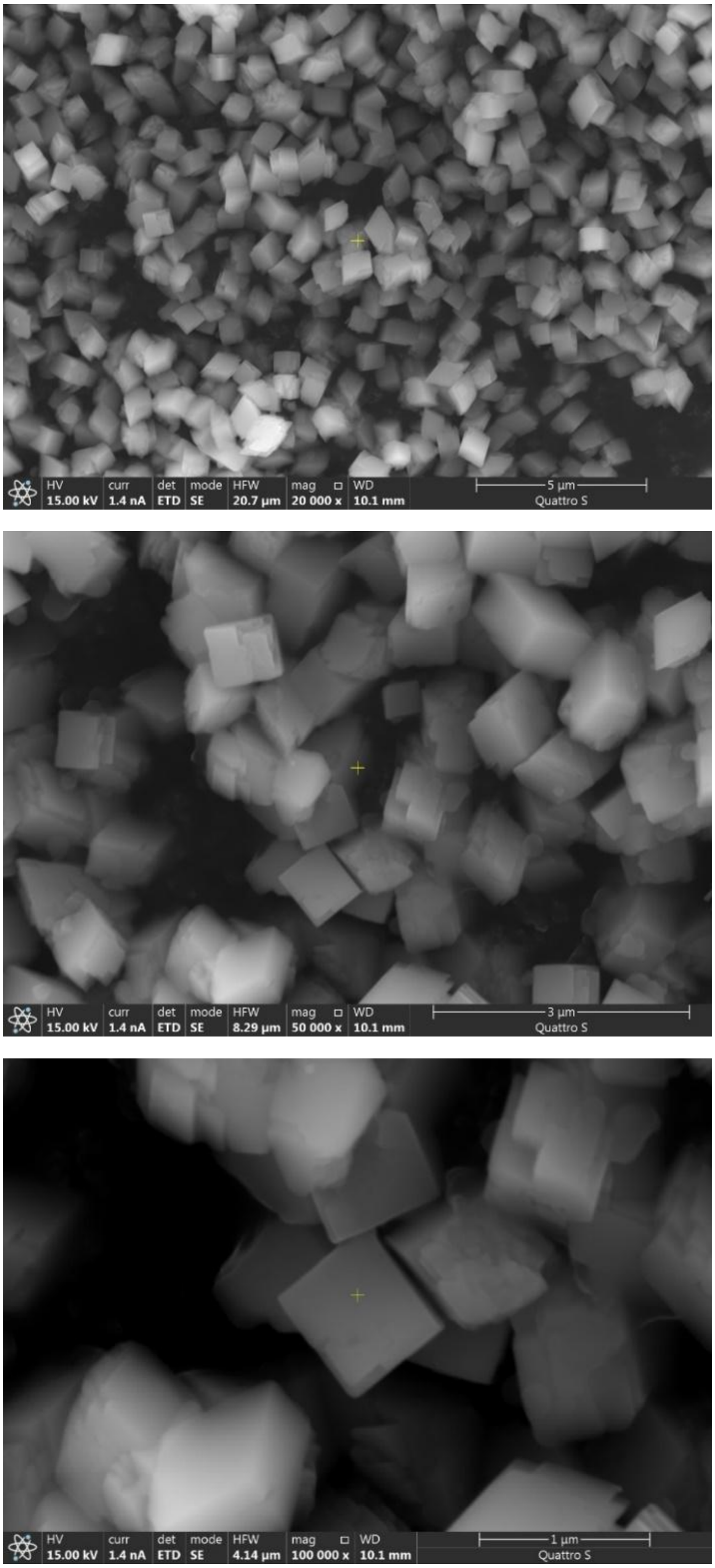

Supplementary Figure 2. SEM micrographs of powder $NaVPO_4F$ at different magnifications of 20kX, 50kX, 100kX.

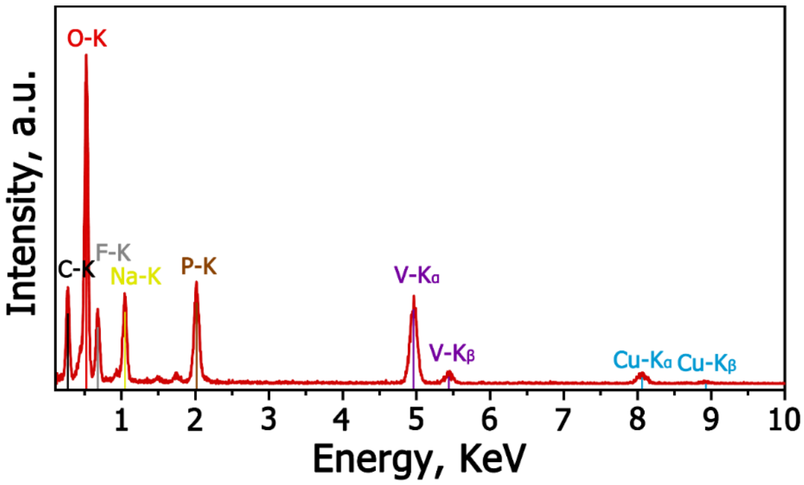

Supplementary Figure 3. Representative TEM-EDX spectrum of powder $NaVPO_4F$ showing the presence of fluorine.

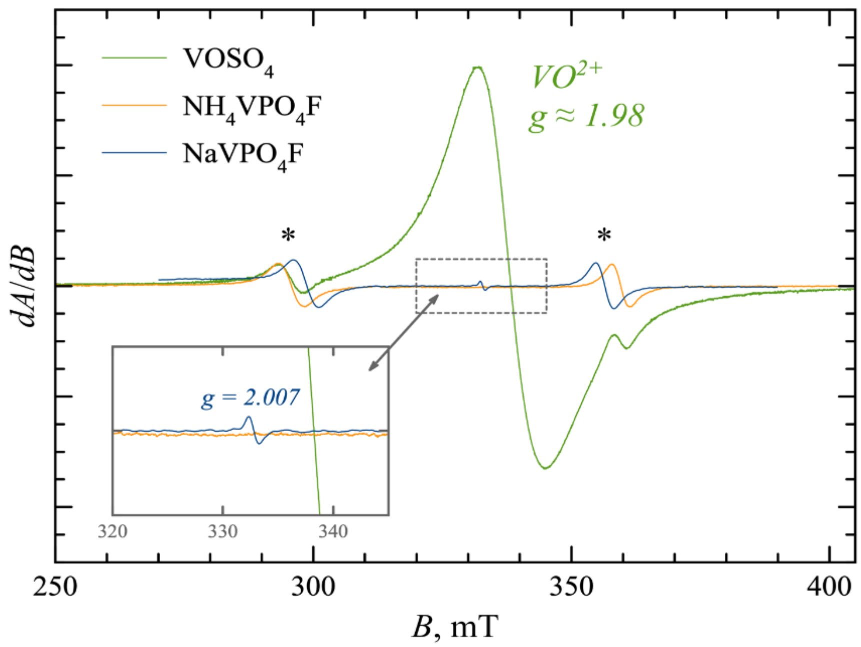

Supplementary Figure 4. EPR spectra of NH_4VPO_4F and $NaVPO_4F$ as compared to the one of $VOSO_4$. Absence of a characteristic VO^{2+} signal evidences the V^{3+} oxidation state in both NH_4VPO_4F and $NaVPO_4F$. (V^{3+} being a non-Kramers ion with two unpaired electrons, S = 1, gives no signal in conventional X-band EPR spectroscopy). Minor signal at *g* = 2.007 observed in the spectrum of $NaVPO_4F$ is due to some carbonaceous impurity, presumably originating from thermal decomposition of sodium glutamate used in the synthesis. Two signals marked with an asterisk come from a crystal of synthetic ruby mounted in the resonator cavity as an internal reference.

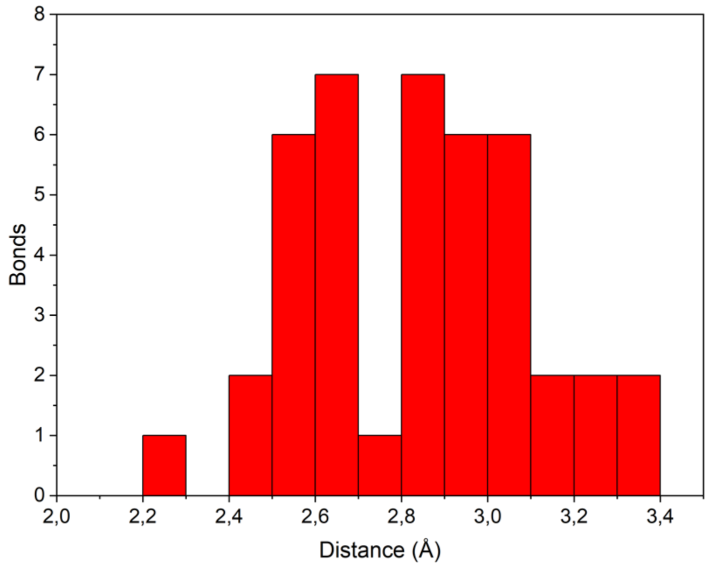

Supplementary Figure 5. Total Na-O/F bond lengths distribution for $NaVPO_4F$.

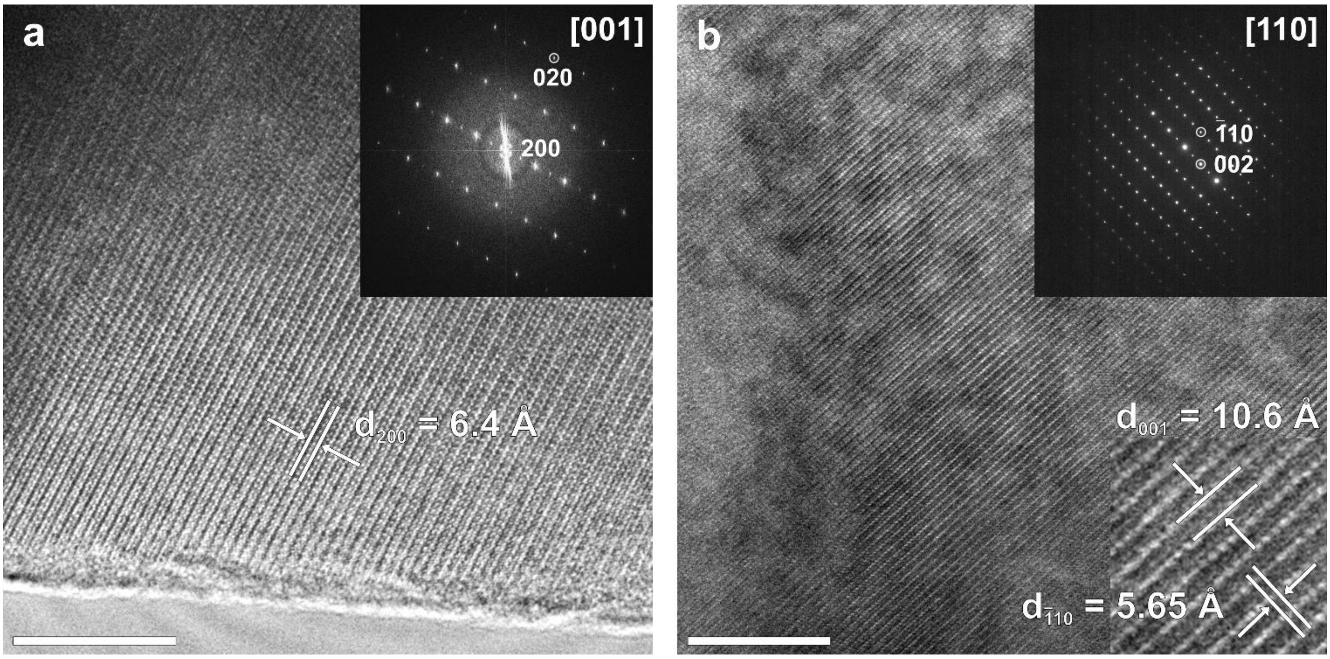

Supplementary Figure 6. HRTEM images for powder $NaVPO_4F$: **a** along the [001] direction, scale bar 10 nm, the inset is a FFT image, **b** along the [110] direction, scale bar 20 nm, the inset is the [110] ED pattern

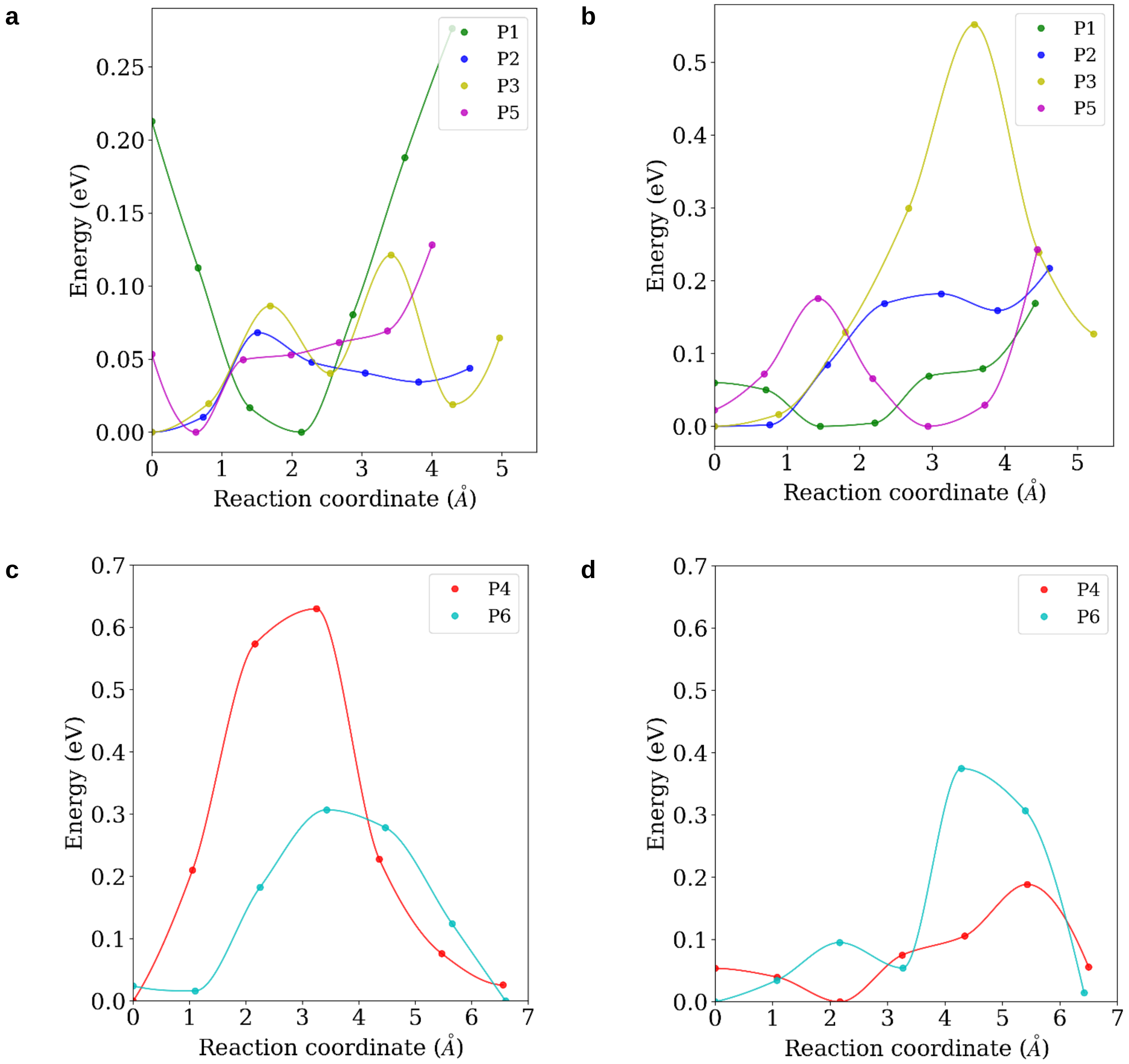

Supplementary Figure 7. DFT-NEB calculated energy profiles for the Na–Na migration pathways in fully inserted state **a**, **c** and fully deinserted state **b**, **d** of $NaVPO_4F$

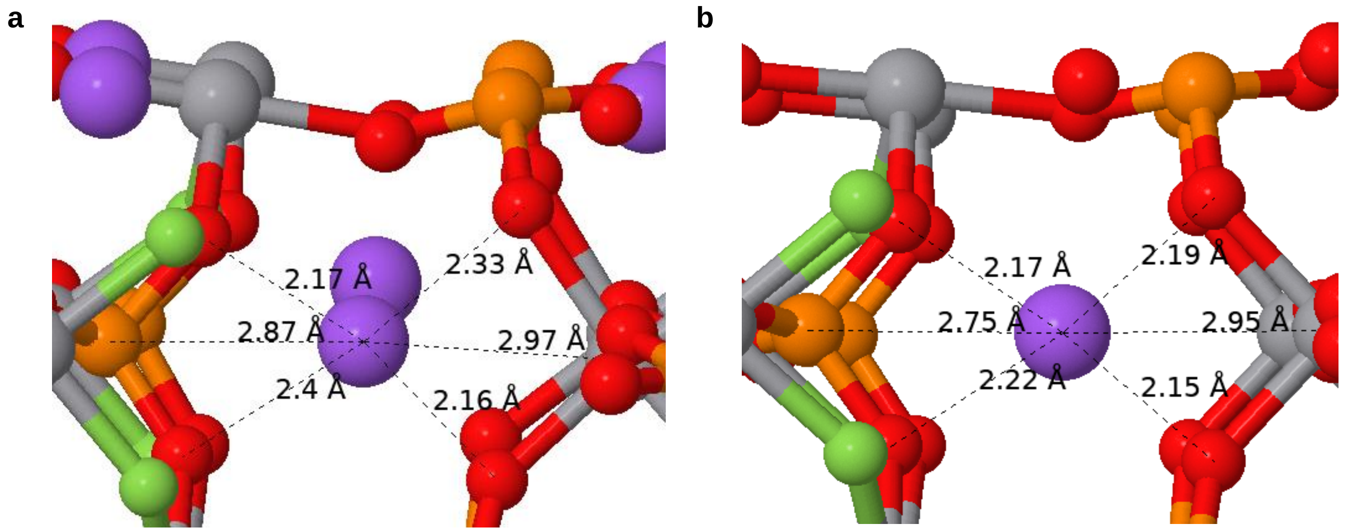

Supplementary Figure 8. The saddle point position of Na for the P3 pathway in **a** $NaVPO_4F$ and **b** VPO_4F. Na, V, O, P and F atoms are shown in violet, gray, red, orange, and green colors respectively. The interatomic distances are shown with dashed lines. The short four distances to oxygen atoms are forming a rectangular window. Two Na-O distances are much shorter in VPO_4F than those in $NaVPO_4F$: 2.22 and 2.19 Å vs. 2.4 and 2.33 Å respectively.

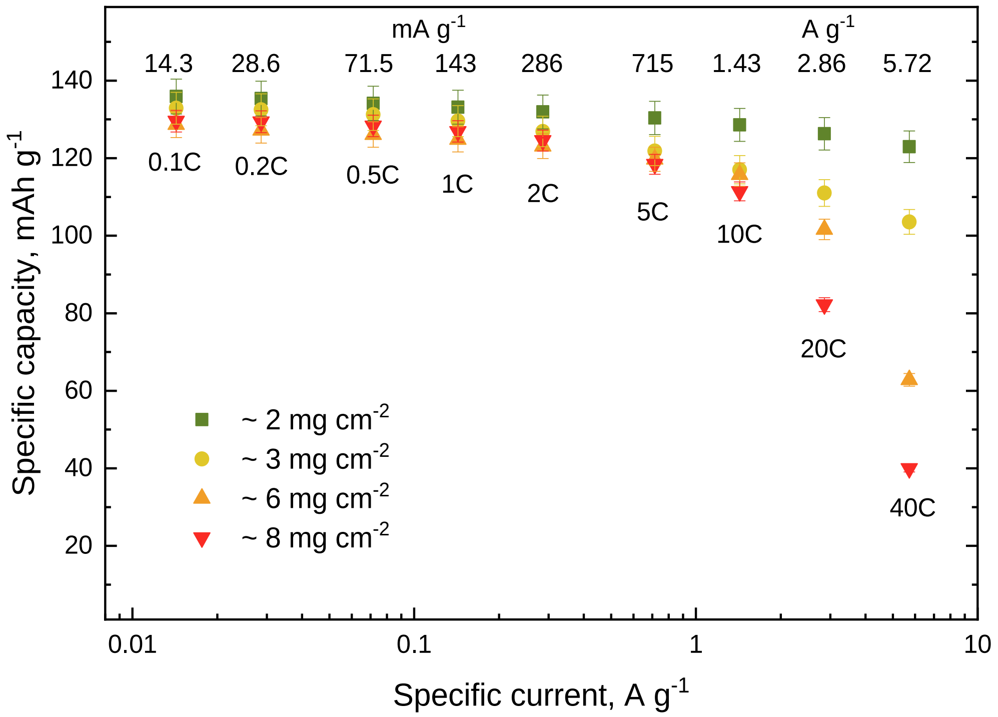

Supplementary Figure 9. The discharge capacities of $NaVPO_4F$/C within the 2.0-4.5 V vs. Na^+/Na potential range at different active material and at various specific currents from 0.1 C (14.3 mA g^{-1}) to 40C (5.72 A g^{-1}). Measurements performed on composite electrodes with 80% active material, 10% Super C carbon black and 10% PVDF at 22±1 °C. The specific capacity was calculated based on the $NaVPO_4F$ material mass. Dots corresponds to the first cycle capacities within the 5 consecutive cycles at each specific current. Error bars are given. Cell configuration: Na||$NaVPO_4F$. The electrolyte used is 1M $NaPF_6$ in EC:PC:FEC (47.5:47.5:5 vol.).

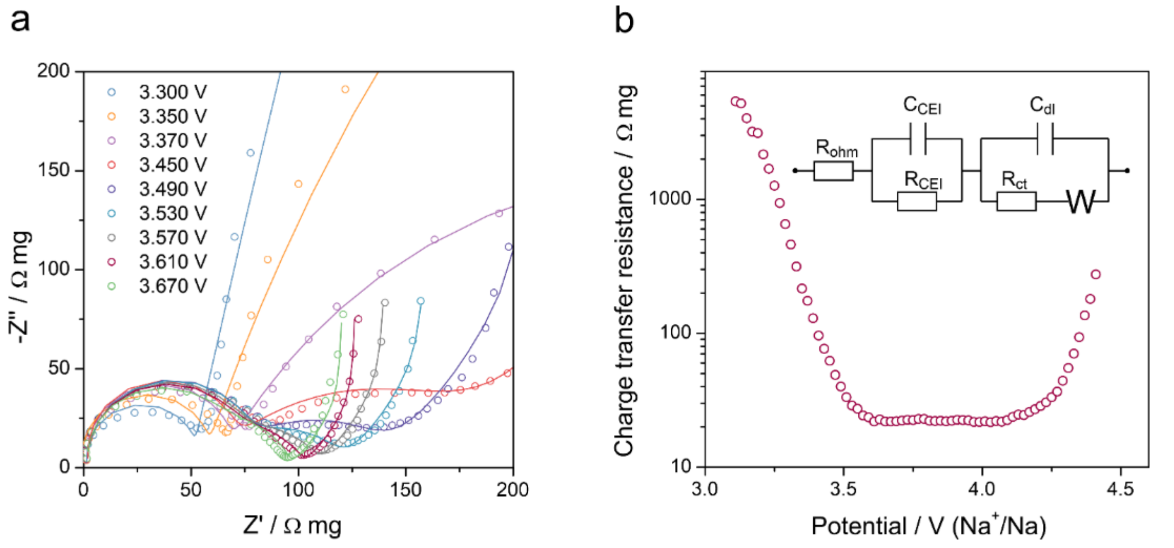

Supplementary Figure 10. a Impedance spectra of the $NaVPO_4F$ electrodes in 1M $NaPF_6$ EC/PC + 5% FEC electrolyte. Symbols are the experimental data, lines are the fits to the equivalent circuit.
b The variation of charge transfer resistance with potential. Insert shows equivalent circuit used to fit the experimental spectra: R_{ohm} – ohmic resistance of the electrolyte; C_{CEI}, R_{CEI} – capacitance and resistance of surface layers; C_{dl} and R_{ct} – double layer capacitance and charge transfer resistance; W – Warburg element for spherical finite-space diffusion. The resistances are normalized to the active mass of the electrode (1.2 mg of active material). EIS data were registered after 3 CV cycles in the potential range 3.0-4.5 V (vs. Na^+/Na) at 0.2 mV s^{-1}. All the measurements were performed at 22±1 °C. Cell configuration: Na||$NaVPO_4F$. In all cases the electrolyte used is 1M $NaPF_6$ in EC:PC:FEC (47.5:47.5:5 vol.)

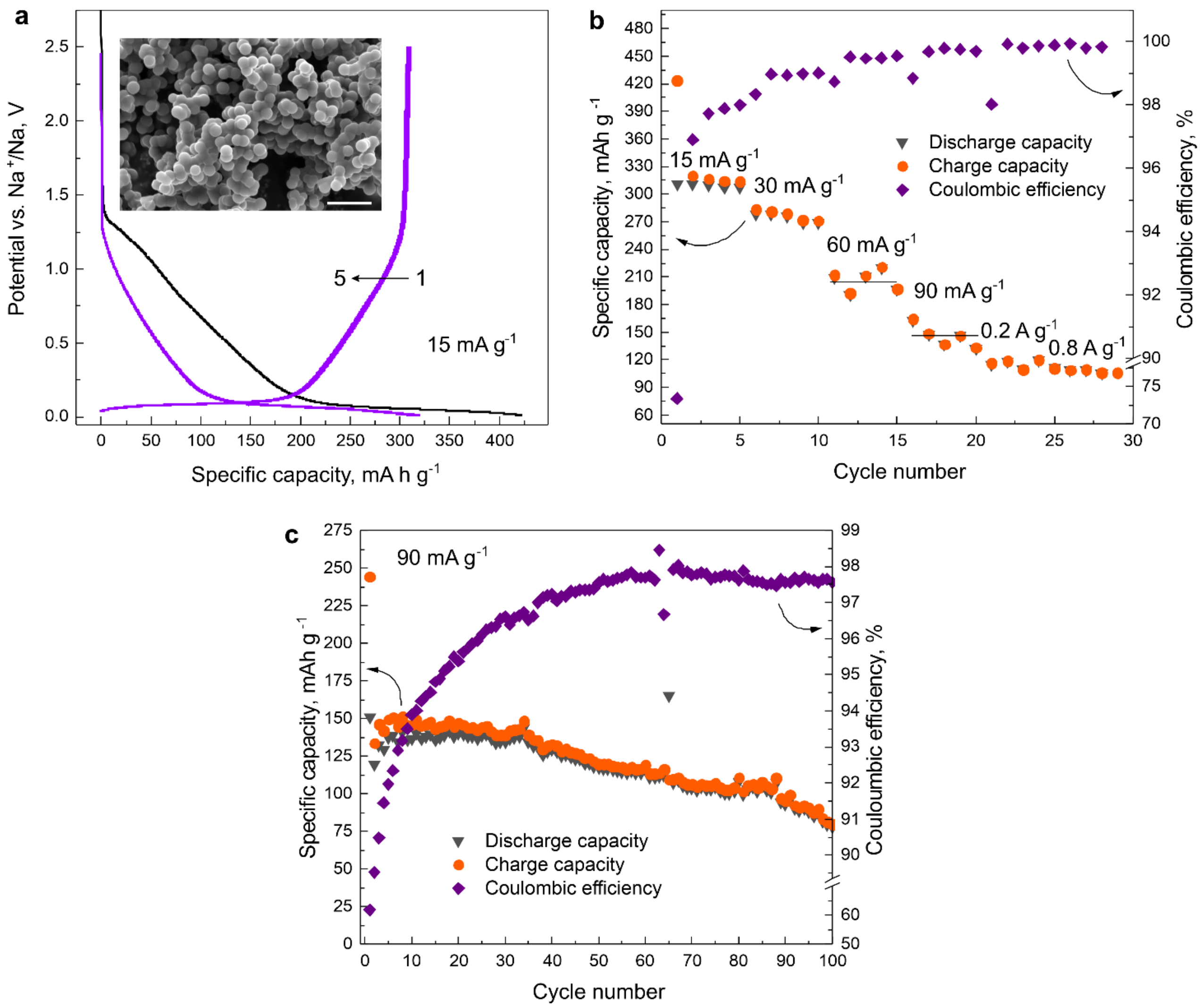

Supplementary Figure 11. **a** Galvanostatic curves for Na||HC cells at 15 mA g^{-1}. Inset: SEM micrograph of the as-prepared powder HC. The scale bar is 2μm. **b** Specific capacities vs. cycle number during at different specific currents. **c** Extended cycling of HC at 90 mA g^{-1} rate. In all cases the electrolyte used is 1M $NaPF_6$ in EC:PC:FEC (47.5:47.5:5 vol.)

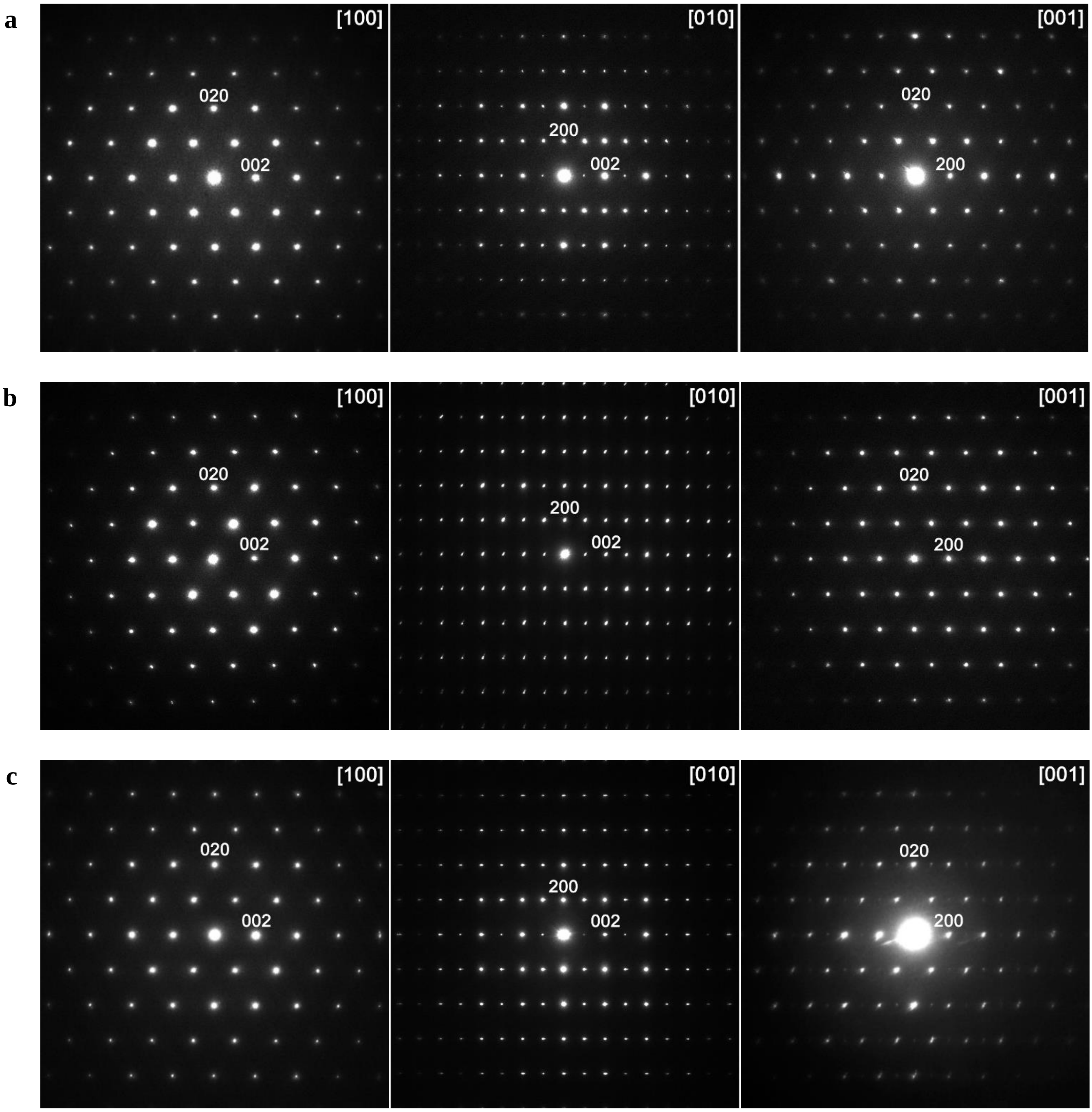

Supplementary Figure 12. Ex situ electron diffraction patterns of the harvested $NaVPO_4F$ electrodes charged to 3.6 V (**a**), 4.5 V (**b**) and discharged to 2.0 V after charging to 4.5 V (**c**).

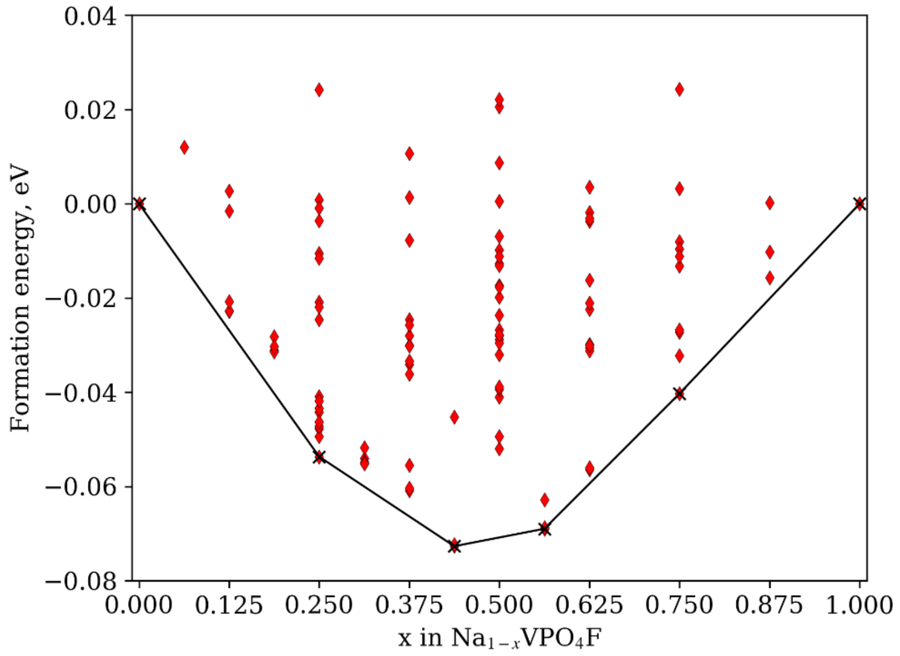

Supplementary Figure 13. Formation energies calculated using the DFT + U method in the $Na_{1-x}VPO_4F$ system coupled with the clusters expansion method to predict possible ground state structures at intermediate concentrations. Cross-marks indicate stable phases.

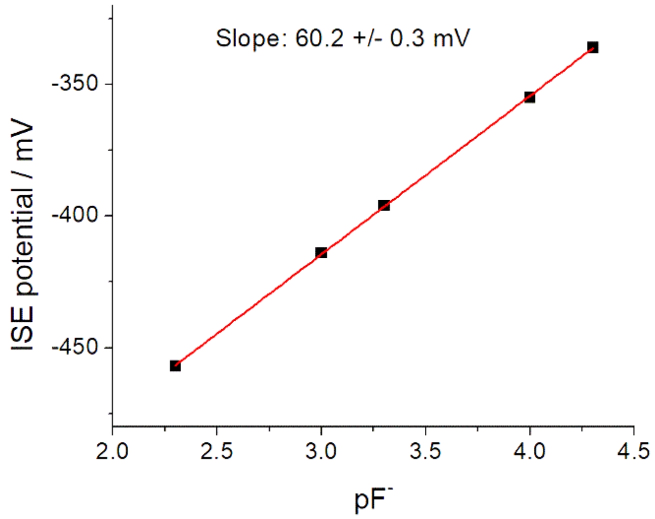

Supplementary Figure 14. Calibration curve showing ion-selective electrode response vs. the logarithm of F^- concentration.

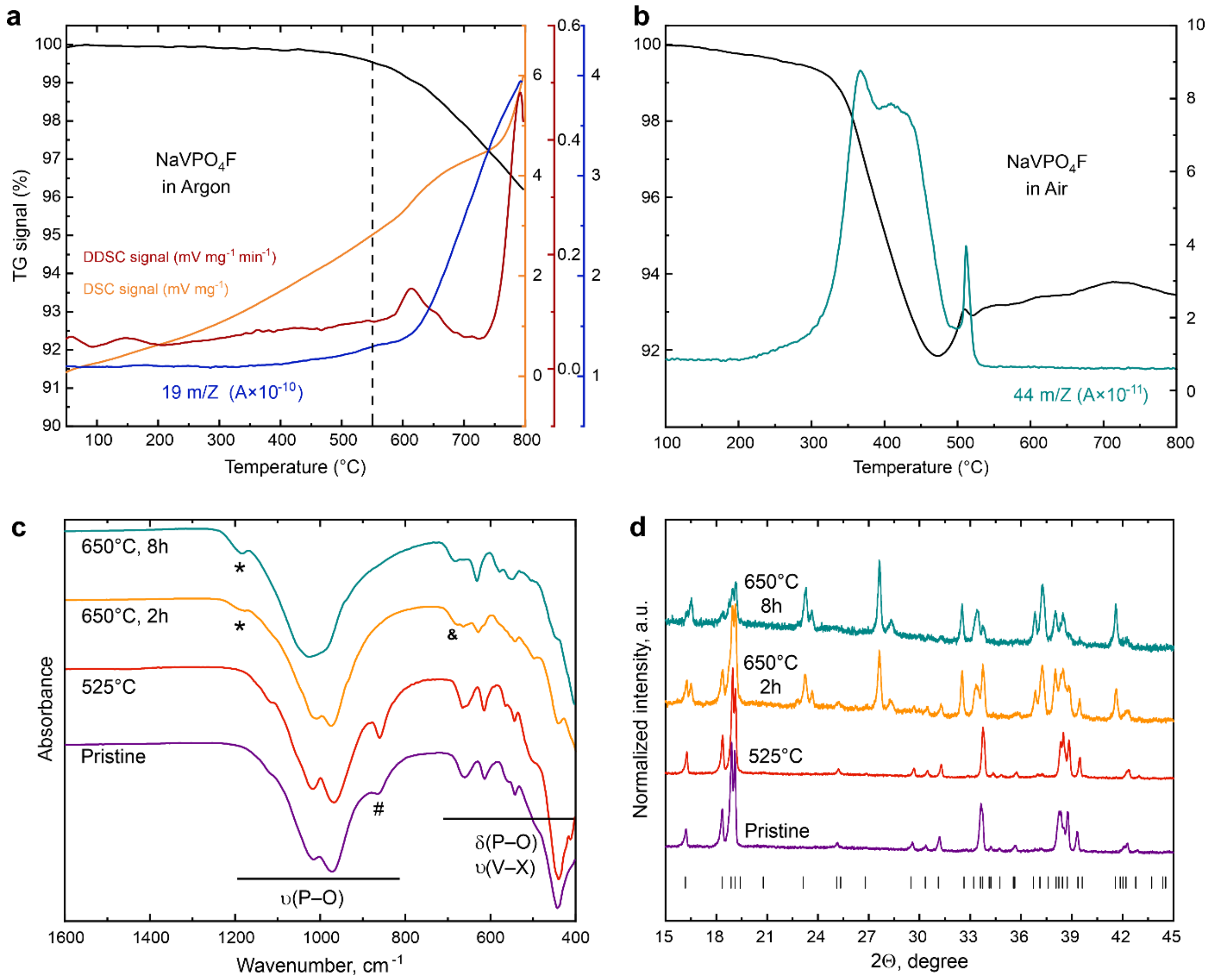

Supplementary Figure 15. TG-MS curves measured in argon for pristine powder $NaVPO_4F$ (**a**) and for carbon-coated powder $NaVPO_4F/C$ (**b**). The m/z 19 curve corresponds to signal from fluoride ion, m/z 44 – CO_2. **c** Temperature-dependent FTIR spectra with corresponding XRD patterns (**d**). The *, $^{\&}$ and $^{\#}$ signs mark the changed or appeared bands. The Bragg's peaks on the XRD pattern are related to the powder $NaVPO_4F$.

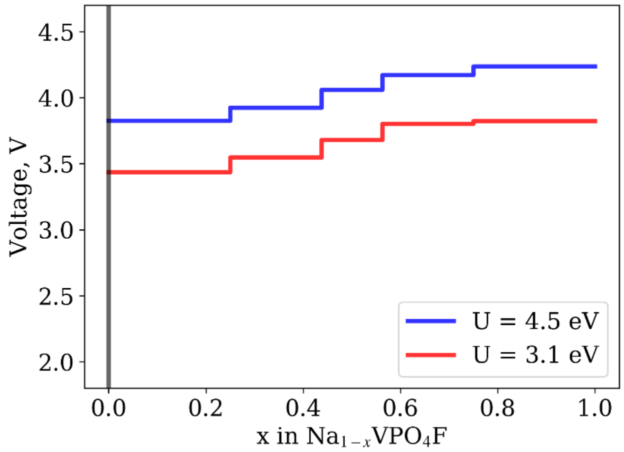

Supplementary Figure 16. Calculated voltage profile based on the ground state phases obtained from the convex hull for two *U* values.

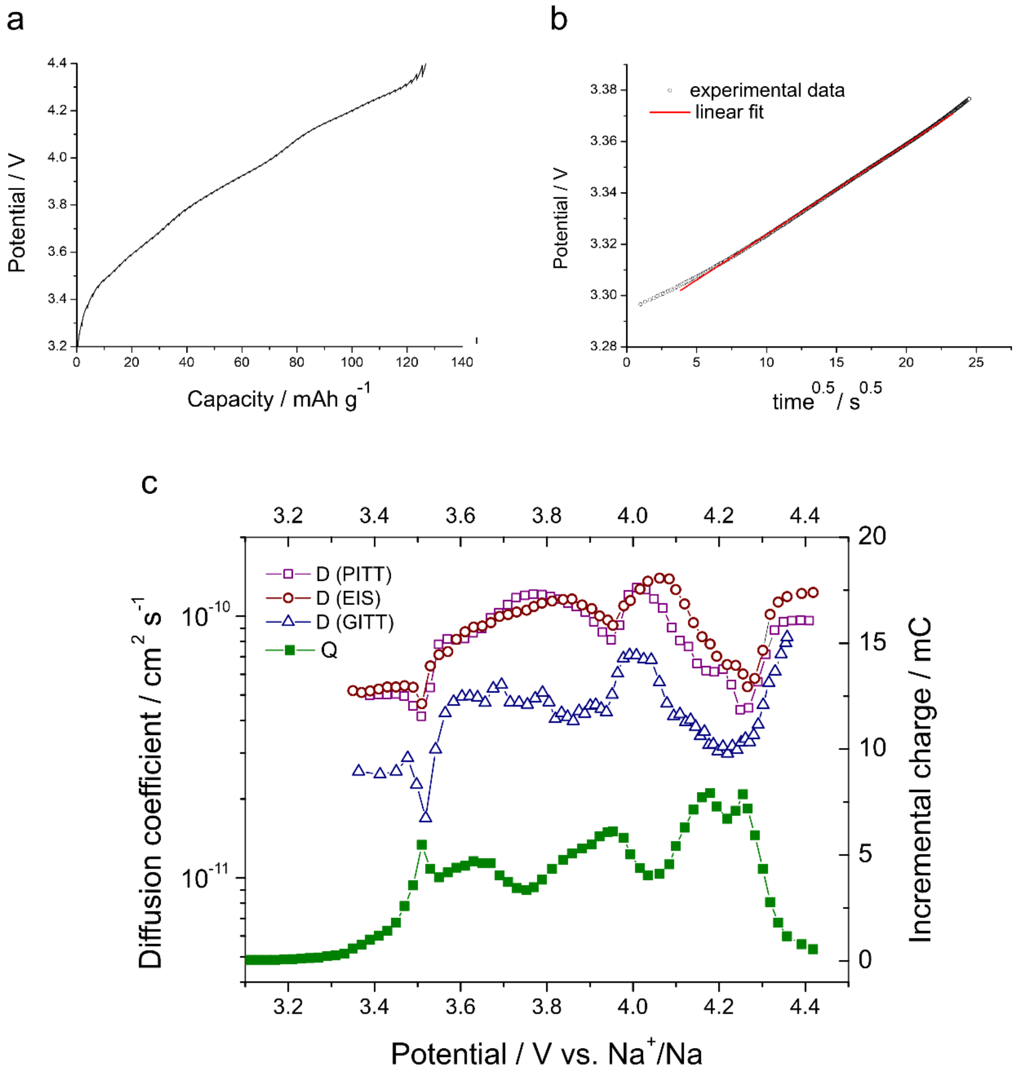

Supplementary Figure 17. a GITT titration curve and **b** representative potential vs. square root of time plot during the charging pulse. **c** Diffusion coefficients, determined from GITT, PITT and EIS. The GITT-pulse duration was 10 min with a current corresponding to 0.1 C (14.3 mA g^{-1}). After each titration step, the cell was allowed to relax for 2 h. This procedure was repeated until a cut-off potential of 4.4 V was reached. PITT and impedance measurements were done in three-electrode cells to ensure precise control of the working electrode potential. Three-electrode cells were stainless steel coin-type cells with sodium disk as a counter electrode on a Cu current collector and a $NaVPO_4F$/C working electrode on an Al current collector. Reference electrodes were fabricated by charging the $Na_3V_2(PO_4)_3$ electrodes (active material:carbon Super-C:pVdF ratio 8:1:1 by mass) as described in [1] to reach the state-of-charge of 0.5 and placed on a stainless steel ring, separated from the counter and working electrodes by PTFE stoppings and glass fiber separators. The potential of the reference electrode was 3.370 V vs. Na^+/Na (the potential of a two-phase transition which accompanies the $V^{4+/3+}$ redox in $Na_3V_2(PO_4)_3$). All the reported potentials were recalculated into the Na^+/Na scale. Measurements performed on a composite electrode with 80% active material, 10% Super P carbon black and 10% PVDF. All the measurements were performed at 22±1 °C). In all cases the electrolyte used is (1M $NaPF_6$ in EC:PC:FEC (47.5:47.5:5 vol.). Specific capacity refers to the mass of the active material of the electrode.

Supplementary Table 1. Fractional atomic coordinates and occupancies* for $NaVPO_4F$ (space group $Pna2_1$).

Atom	Wyckoff position	*x/a*	*y/b*	*z/c*	Occupancy
Na1	4*a*	0.435(4)	0.813(6)	0.057(5)	0.247(21)
Na2	4*a*	0.3556(22)	0.789(4)	0.027(4)	0.445(21)
Na3	4*a*	0.0901(21)	0.391(3)	0.729(3)	0.409(14)
Na4	4*a*	0.372(10)	0.217(23)	0.489(19)	0.092(17)
Na5	4*a*	0.1095(11)	0.7625(21)	0.7960(15)	0.807(14)
V1	4*a*	0.38257(30)	0.5130(9)	0.7538(14)	1.000
V2**	4*a*	0.2410(5)	0.2634(10)	0	1.000
P1	4*a*	0.4934(9)	0.3362(9)	0.0108(15)	1.000
P2	4*a*	0.1881(5)	0.4961(21)	0.2575(18)	1.000
O1	4*a*	0.4936(16)	0.4640(29)	0.8871(19)	1.000
O2	4*a*	0.5161(13)	0.5013(29)	0.1150(20)	1.000
O3	4*a*	0.3903(9)	0.2157(29)	0.0163(28)	1.000
O4	4*a*	0.5900(9)	0.1992(26)	0.9904(27)	1.000
O5	4*a*	0.1105(13)	0.3182(22)	0.2941(20)	1.000
O6	4*a*	0.1153(13)	0.6882(20)	0.2395(24)	1.000
O7	4*a*	0.2712(13)	0.5583(28)	0.3571(18)	1.000
O8	4*a*	0.2558(14)	0.499(4)	0.1357(17)	1.000
F1	4*a*	0.2864(10)	0.5253(29)	0.6160(17)	1.000
F2	4*a*	0.2514(10)	0.4695(30)	0.8689(16)	1.000

* - Atomic displacement parameters were fixed to 0.01 $Å^2$.
** - The z coordinate was fixed to 0 for the V2 atom to define the origin.

Supplementary Table 2. Selected bond lengths for $NaVPO_4F$.

Atom 1	Atom 2	Distance, Å	Atom 1	Atom 2	Distance, Å
V1	O1	2.035(16)	V2	O3	1.930(14)
V1	O2	1.955(16)	V2	O4	1.947(14)
V1	O5	1.960(15)	V2	O7	2.004(17)
V1	O6	2.041(13)	V2	O8	2.076(19)
V1	F1	1.916(15)	V2	F1	1.968(17)
V1	F2	2.090(13)	V2	F2	1.901(15)
P1	O1	1.542(6)	P2	O5	1.541(6)
P1	O2	1.544(6)	P2	O6	1.533(6)
P1	O3	1.517(6)	P2	O7	1.549(6)
P1	O4	1.517(6)	P2	O8	1.557(6)

Supplementary Table 3. Parameters of EIS data fitting for $NaVPO_4F$ electrode. In the fitting procedure, CPE elements were used instead of capacitances (ϕ_{CEI}, ϕ_{dl} – constant phase exponent for C_{CEI} and C_{dl}). R_{diff} (resistance of diffusion step) and C_{int} (capacitance of diffusion step) are the parameters of finite Warburg element.

E, V	R_{ohm}, Ω	R_{ct}, Ω	R_{CEI}, Ω	R_{diff}, Ω	C_{int}, F	C_{CEI}, μF	ϕ_{CEI}	C_{dl}, mF	ϕ_{dl}
3.300	$5.2\cdot10^{-3} \pm 1\cdot10^{-4}$	5321 ± 925	61.41 ± 0.06	3143 ± 546	0.0019 ± 0.0002	3.93 ± 0.49	1.04 ± 0.01	0.84 ± 0.04	0.90 ± 0.02
3.350	$4.3\cdot10^{-3} \pm 1\cdot10^{-4}$	1614 ± 53	62.04 ± 0.07	2456 ± 348	0.0096 ± 0.0006	3.21 ± 0.16	1.03 ± 0.01	0.80 ± 0.02	0.85 ± 0.01
3.370	$5.3\cdot10^{-3} \pm 2\cdot10^{-4}$	256 ± 13	62.53 ± 0.01	2085 ± 438	0.040 ± 0.006	2.90 ± 0.06	1.04 ± 0.02	1.15 ± 0.06	0.84 ± 0.02
3.450	$3.7\cdot10^{-3} \pm 1\cdot10^{-4}$	95.1±2.4	68.13 ± 0.08	639 ± 84	0.094 ± 0.009	3.14 ± 0.02	1.02 ± 0.02	1.19 ± 0.03	0.726 ± 0.004
3.490	$5.6\cdot10^{-3} \pm 2\cdot10^{-4}$	63.0 ± 1.9	69.04 ± 0.46	408 ± 77	0.183 ± 0.027	3.11 ± 0.02	1.02 ± 0.02	0.86 ± 0.03	0.750 ± 0.004
3.530	$8.2\cdot10^{-3} \pm 3\cdot10^{-4}$	48.1 ± 1.1	66.29 ± 0.06	229 ± 31	0.222 ± 0.020	2.86 ± 0.01	1.03 ± 0.01	0.50 ± 0.1	0.786 ± 0.004
3.570	$6.2\cdot10^{-3} \pm 5\cdot10^{-4}$	44.3 ± 0.9	59.72 ± 0.16	182 ± 18	0.211 ± 0.012	2.65 ± 0.01	1.04 ± 0.02	0.200 ± 0.004	0.856 ± 0.001
3.610	$7.8\cdot10^{-3} \pm 3\cdot10^{-4}$	42.5 ± 0.6	54.14 ± 0.04	151 ± 13	0.229 ± 0.010	2.55 ± 0.01	1.05 ± 0.01	0.120 ± 0.002	0.897 ± 0.003
3.670	$4.7\cdot10^{-3} \pm 1\cdot10^{-4}$	34.5 ± 0.5	55.53 ± 0.11	137 ± 10	0.222 ± 0.008	2.90 ± 0.06	1.03 ± 0.02	0.100 ± 0.003	0.940 ± 0.008

Supplementary note 1

DFT-NEB study

The DFT+U optimized lattice parameters for the indexed unit cell (*a* = 12.85 Å, *b* = 6.34 Å, *c* = 10.76 Å, V = 877.2 Å^3) are in a good agreement with the experiment.

To study the diffusion channels in more detail we calculated migration barriers with the DFT-NEB method for six independent paths (P1-P6). The NEB-optimized trajectories of short paths (P1-P3 and P5) and corresponding energy profiles for migration are provided in Figure 1h and Supplementary Figure 7. The calculated migration barriers for P1, P2, P3 and P5 are 0.3, 0.1, 0.1, and 0.15 eV, respectively. Interestingly, the diffusion migration barrier along the helical channel P1-P2 responsible for percolation along the *c* direction is 0.1 eV larger for Na^+ vacancy than that for K^+ vacancy in $KVPO_4F$. On the other hand, the migration along P3 and P5 are significantly easier for Na^+ vacancy (0.1 and 0.15 eV) than that for the K^+ one (1.1 and 0.3 eV). This allows a percolating diffusion along the *a* (P1+P5) and *b* (P2+P3) directions with only 0.3 eV and 0.1 eV migration barriers, respectively. The energy profiles for migration are given in Supplementary Figure 6. After creating one Na^+ vacancy in $NaVPO_4F$ the minima are not located at or close to the initial sites but distributed along the migration path, which agrees with the experimentally observed pronounced disorder of Na positions.

The migration barriers for a Na^+ ion in the fully deinserted state (□VPO_4F) are 0.2, 0.2, 0.55, and 0.25 eV for the P1, P2, P3, and P5 paths, respectively. This means that the migration along the *a*, *b,* and *c* axes require 0.25, 0.55, and 0.2 eV, respectively. Hence, the migration along *b* becomes slightly hindered in course of Na deinsertion, while the hopping along the *a* and *c* directions gets easier. This emphasizes that $NaVPO_4F$ should demonstrate good ion mobility in the whole range of Na concentrations, which is confirmed further by diffusion coefficient measurements and rate capabilities tests. The noticeable variation of migration barriers for the P3 path is related to the necessity of the cation to move through the rectangular window composed of four oxygen atoms (Supplementary Figure 8). The window is significantly compacted in the case of the empty "$VPOF_4$" framework explaining the increase of the migration barrier.

The two long paths, P4 (along *a*) and P6 (along *b*) (Supplementary Figure 7) which are 0.6 and 0.3 eV for the fully inserted state, and 0.2 and 0.4 eV for the fully deinserted state respectively. Therefore, these paths can further accelerate the diffusion along the *b* and *a* directions in $NaVPO_4F$ and VPO_4F. Interestingly, the DFT-derived picture of migration pathways resembles that of the BVEL analysis, with the most hindered P4 path expectedly being neglected by BVEL.

For further understanding of the structural transformation during charge/discharge and interpreting of the *operando* SXPD results and CV we calculated a convex hull for the $Na_{1-x}VPO_4F$ system using DFT+U and cluster expansion methods. The DFT+U optimized lattice parameters for the indexed unit cell (*a* = 12.85 Å, *b* = 6.34 Å, *c* = 10.76 Å, V = 877.2 Å^3) are in a good agreement with the experiment. The

convex hull provided in Supplementary Figure 13. It shows stabilization of phases at four vacancy concentrations: 0.25, 0.44, 0.56, and 0.75, but not at 0.5 as opposed to K-containing $KMPO_4F$ (M = Ti, V). At intermediate concentrations of 0.125, 0.313, 0.375, 0.625, and 0.875 the phases are only by 3-6 meV fu^{-1} above the convex hull. At room temperature, such a driving force is insufficient for a phase separation, as the configurational entropy contribution of the Na/vacancy mixing is in the order of −12.5 meV fu^{-1}, which is further doubled due to the mixing of V^{3+}/V^{4+}. The $Pna2_1$-to-*Pnan* transition observed at the beginning of deintercalation is most probably an order/disorder transition occurring in the Na sublattice, where partly ordered $NaVPO_4F$ is in equilibrium with fully disordered $Na_{1-x}VPO_4F$ (x ~ 0.15). This transition and its electrochemical response strongly remind the well-known order-disorder transitions in $LiCoO_2$ [2]. It is important that the disorder in $Na_{1-x}VPO_4F$ takes place over several Na positions located along the diffusion channels. Therefore, the provided convex hull, which was constructed using cluster expansion for two Na positions in the idealized KTP-type $NaVPO_4F$ structure, might have omitted some low-temperature phases with intermediate Na positions. For x > 0.15 the *Pnan* $Na_{1-x}VPO_4F$ operates via a single solid-solution regime. However, in contrast to a regular solid solution, where Na positions have completely random coordination within the Na sublattice, the local Na/vacancy and V^{3+}/V^{4+} ordering might give rise to several ensembles of positions with a similar short-range coordination and Gaussian-like distribution of the de/insertion electrode potentials. The existence of such ensembles of positions is emphasized by the three wide peaks on the dQ/dE plot (Figure 2a, inset), however, these orderings are indistinguishable in *operando* XRD due to their short-range, local nature.

The average insertion potential for $NaVPO_4F/VPO_4F$ is 3.65 V for the *U* value of 3.1 eV [3]. We found that to reproduce the experimentally observed average insertion potential the *U* value should be increased up to 4.5 eV. However, for both U values the slope of the calculated voltage profile is narrower compared to the experiment (Supplementary Figure 16). A more accurate simulation of the experimental voltage profile in this cathode material may require using more sophisticated computational approaches *e.g.* using hybrid functionals.

Supplementary note 2

Thermal behavior of $NaVPO_4F$ by TG and temperature-dependent FTIR coupled with XRD

The reported orthogonal KTP-type $NaVPO_4F$ shows thermal stability up to 525-550 °C as was validated by a joint TG-MS analysis (Supplementary Figure 15a). To further understand the thermal behavior of the materials we performed joint temperature-dependent XRD and FTIR analysis (Supplementary Figure 15c, d). According to the obtained data, the material undergoes a decomposition reaction at temperatures higher than 550°C, which is clearly shown by the appearance additional reflections and bands at XRD and FTIR correspondingly as well as by a peak in the first derivative curve of the DSC signal. Also, at the FTIR spectrum a shift of the vibrational modes for the phosphate anion can be evidenced at 650°C which is getting more pronounced at longer annealing time. The main decomposition products were found to be NASICON-type $Na_3V_2(PO_4)_3$ and $Na_3V_2(PO_4)_2F_3$ which corroborates well with the fluorine removal from the system as detected by mass-spectrometry (Supplementary Figure 15a). However, at 525°C the XRD pattern and FTIR spectrum are in a good agreement with those of the pristine as-prepared material (Supplementary Figure 15c, d, respectively).

Supplementary note 3

GITT measurements

We also performed GITT experiment to determine diffusion coefficients (the information is collected in Supplementary Figure 17). The apparent diffusion coefficients, estimated from GITT, appear to be lower than those derived from PITT and EIS (Supplementary Figure 17). This is not surprising, as the procedure of Weppner and Huggins assumes no charge transfer limitations, while the transients for $NaVPO_4F$ are affected by the finite rate of surface reaction. PITT and EIS methods take into account the limitations imposed by slow kinetics, which makes the apparent diffusion values derived from these methods more reliable. Yet, since the diffusion coefficients from PITT and GITT differ only by the factor of 2, we can state that the effect of slow interfacial kinetics is minor.

Supplementary references

[1] Anishchenko, D. V., Zakharkin, M. V., Nikitina, V. A., Stevenson, K. J. & Antipov, E. V. *Electrochim. Acta* **354**, 136761 (2020).

[2] J. N. Reimers and J. R. Dahn *J. Electrochem. Soc*. **139** 2091-2097 (1992).

[3] S. S. Fedotov, A. Sh. Samarin, V. A. Nikitina et al. *J. Mater. Chem. A*, **6**, 14420-14430 (2018)
